# Bacteria-Induced Uroplakin Signaling Mediates Bladder Response to Infection

**DOI:** 10.1371/journal.ppat.1000415

**Published:** 2009-05-01

**Authors:** Praveen Thumbikat, Ruth E. Berry, Ge Zhou, Benjamin K. Billips, Ryan E. Yaggie, Tetiana Zaichuk, Tung-Tien Sun, Anthony J. Schaeffer, David J. Klumpp

**Affiliations:** 1 Department of Urology, Feinberg School of Medicine, Northwestern University, Chicago, Illinois, United States of America; 2 Department of Microbiology-Immunology, Feinberg School of Medicine, Northwestern University, Chicago, Illinois, United States of America; 3 Departments of Cell Biology, Dermatology, Pharmacology and Urology, New York University School of Medicine, New York, New York, United States of America; University of British Columbia, Canada

## Abstract

Urinary tract infections are the second most common infectious disease in humans and are predominantly caused by uropathogenic *E. coli* (UPEC). A majority of UPEC isolates express the type 1 pilus adhesin, FimH, and cell culture and murine studies demonstrate that FimH is involved in invasion and apoptosis of urothelial cells. FimH initiates bladder pathology by binding to the uroplakin receptor complex, but the subsequent events mediating pathogenesis have not been fully characterized. We report a hitherto undiscovered signaling role for the UPIIIa protein, the only major uroplakin with a potential cytoplasmic signaling domain, in bacterial invasion and apoptosis. In response to FimH adhesin binding, the UPIIIa cytoplasmic tail undergoes phosphorylation on a specific threonine residue by casein kinase II, followed by an elevation of intracellular calcium. Pharmacological inhibition of these signaling events abrogates bacterial invasion and urothelial apoptosis *in vitro* and *in vivo*. Our studies suggest that bacteria-induced UPIIIa signaling is a critical mediator of bladder responses to insult by uropathogenic *E. coli*.

## Author Summary

Urinary tract infections (UTI) are the second most common infectious disease in humans and are predominantly caused by uropathogenic *E. coli* (UPEC). *In vitro* and *in vivo* studies have demonstrated that UPEC induce several responses in the bladder, including inflammation, rapid onset of bladder cell death, and bacterial invasion of bladder cells. This last event, invasion, is now also thought to underlie recurrent UTI. Although members of the highly-expressed “uroplakin” proteins serve as bladder receptors for UPEC binding, it was unclear how UPEC binding to uroplakin receptors caused signals within bladder cells that mediate rapid cell death and bacterial invasion. Here, we show that another uroplakin, uroplakin III, which is associated with the receptor transduces signals within the cell in response to UPEC binding. UPEC causes elevated calcium within bladder cells, and this elevation requires phosphorylation of uroplakin III by a specific kinase. Blocking these events blocks both bladder cell death and bacterial invasion of bladder cells by UPEC. Thus, uroplakin III is the mediator of key events in UTI pathogenesis.

## Introduction

Urinary tract infections (UTIs) are the second most common infectious disease in humans, following respiratory tract infections. Approximately 90% of community-acquired UTIs are caused by uropathogenic *E. coli* (UPEC) [1]. The type 1 pilus is the most common UPEC virulence factor; present in over 90% of clinical UPEC isolates and is required to establish cystitis [2],[3],[4]. Type 1 pili mediate attachment to host cells by virtue of the adhesin protein FimH that occupies the pilus tip [5]. FimH possesses a lectin activity specific for mannosylated proteins that maintains bacterial attachment to the urothelium during urine voiding [6]. Following attachment type-1 pili promote bacterial invasion of urothelial cells, thereby contributing to the formation of intracellular bacterial communities (IBCs) [7],[8]. Host cell actin reorganization, PI3-kinase activation, and host protein tyrosine phosphorylation have all been associated with this invasion process in cell culture models [7]. Recent studies employing both cell culture and murine UTI models suggest that UPEC also commandeer the constitutive endocytic/exocytic machinery of urothelial cells early during infection, where bacteria reside in Rab27b/CD63-positive fusiform vesicles [9]. Invasive bacteria can then exploit the cAMP-regulated exocytic process to re-enter the bladder lumen during bladder distension.

Urothelium responds to UPEC insult by secreting inflammatory cytokines and chemokines. IL-6 and IL-8 are detectable in UTI patient urine, and murine UTI studies show that the recruitment of neutrophils mediates bacterial clearance by phagocytosis (reviewed in [10]). Superficial urothelial cells also undergo rapid apoptosis and are exfoliated into the lumen of the bladder in murine UTI studies, presumably as a host defense mechanism that contributes to bacterial clearance by purging tissue-associated bacteria during voiding [10]. This urothelial apoptotic process is dependent upon the bacterial expression of type 1 pili since its FimH activates classical extrinsic and intrinsic apoptotic cascades [11],[12],[13]. Despite our increased understanding of FimH-induced UPEC invasion and urothelial apoptosis, the signal transducer and downstream second messenger that mediate these two critical events is currently unknown.

The bladder urothelium is a stratified epithelium with a superficial layer of “umbrella” cells that are characterized by a highly specialized apical plasma membrane, the asymmetric unit membrane (AUM). This unique membrane structure is comprised mainly of four integral membrane proteins, the uroplakins (UPs) Ia, Ib, II and IIIa, [14],[15],[16],[17],[18]. The AUM is a component of the permeability barrier that protects underlying tissues from noxious components of urine because UPIIIa knockout mice exhibit both altered AUM structure and defective barrier function [19]. In addition to their roles in AUM structure, UPIa plays an important role in UPEC pathogenesis by serving as the receptor for FimH [20],[21],[22]. UPII and UPIIIa are type-1 transmembrane proteins that undergo obligatory heterodimerization with UPIa and UPIb, respectively, during transport to the apical cell surface [23]. Of the four major uroplakins, UPIIIa alone is predicted to have an appreciable cytoplasmic domain that may function as a signal transducer [17].

Activation of host signal transduction cascades by bacterial attachment is a well-recognized consequence of host-pathogen interactions [24], and immediate urothelial signaling events are associated with UPEC invasion and urothelial cell apoptosis [25],[26]. Recent evidence suggests a possible signaling function for human uroplakins because xUPIII contributes to *Xenopus* sperm-egg fusion [27]. The xUPIII cytoplasmic tail was shown to undergo phosphorylation on a tyrosine residue and result in *Src* kinase-dependent egg activation [27],[28],[29]. This raises the possibility that mammalian UPIIIa also plays a signaling role in the bladder, in addition to its participation in forming the AUM permeability barrier. We hypothesized that human UPIIIa transduces urothelial signals that mediate UPEC pathogenesis. Here, we report a signaling role for the UPIIIa in bacterial invasion of urothelial cells and in UPEC-induced urothelial cell apoptosis. FimH induces phosphorylation of threonine-244g on the UPIIIa cytoplasmic tail by casein kinase II, followed by an increase in intracellular calcium concentration. Pharmacologic inhibition of these events abrogates bacterial invasion and apoptosis both *in vitro* and *in vivo*. Our studies suggest that bacteria-induced UPIIIa signaling is a critical mediator of the pathogenic cascade induced in the host cell and identify a novel therapeutic target for intervention in UTI pathogenesis.

## Results

### Bacterial FimH interacts with the uroplakin receptor complex in cultured human urothelial cells

**Figure 1:**
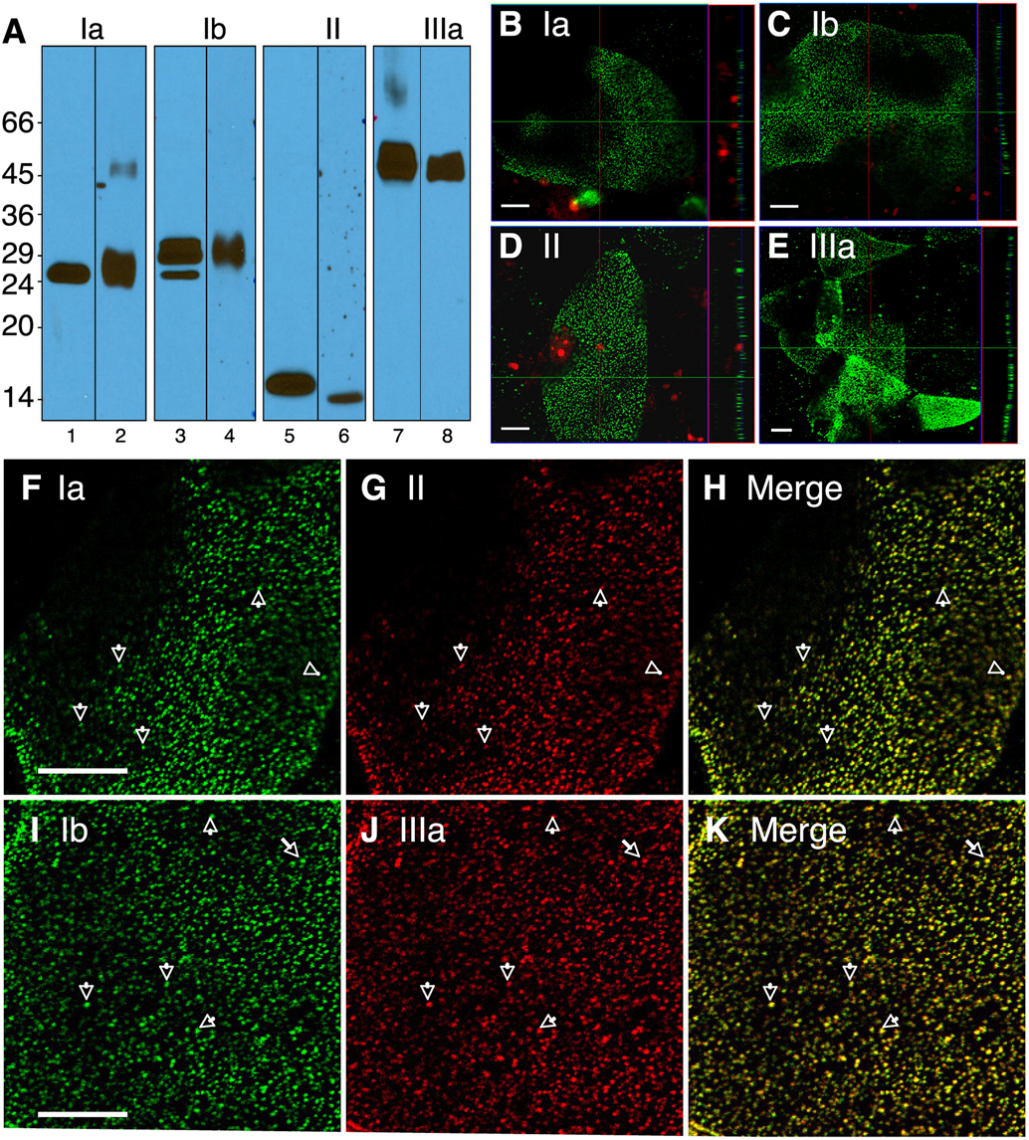
Localization of surface-expressed uroplakins on PD07i cells. (A) 0.1 ug of bovine (lane 1, 3, 5, 7) and human (lane 2, 4, 6, 8) AUM proteins were resolved on 17% SDS-PAGE and transferred onto a nitrocellulose membrane, and detected with monospecific rabbit antisera against Ia (lane 1, 2), Ib (lane 3, 4), II (lane 5, 6), and IIIa (lane 7, 8). All the uroplakins antibodies are monospecific to a single uroplakin band in both bovine and human AUM. The surface expression of uroplakins on PD07i cells were detected using immunofluorescent staining using anti-uroplakins Ia (B), Ib (C), II (D) and IIIa (E) followed with Alexa Fluor 488-conjugated donkey anti-rabbit IgG. Propidium iodide (red) was used to stain nuclei (B–E). Double staining of uroplakins Ia (F) / II (G) / merge (H), and Ib (I) / IIIa (J) / merge (K) showed co-localization of uroplakins on the apical surface of PD07i cells. Arrows mark the co-localization of surface-expressed uroplakins (F–K).

**Figure 2:**
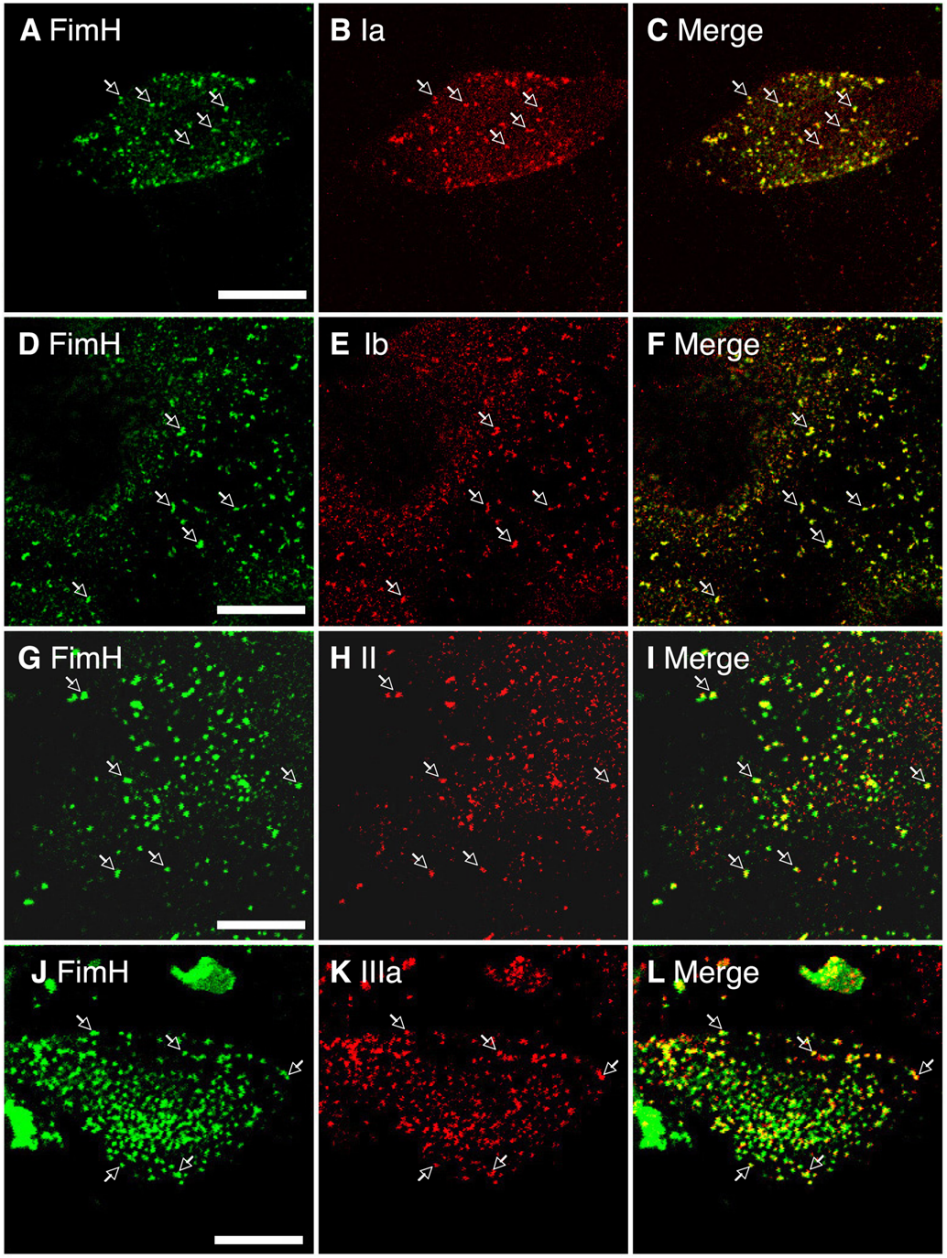
Co-localization of uroplakins and FimH binding sites on PD07i cell surface. The surface-expressed uroplakins on PD07i cells were detected using antisera against individual uroplakins Ia (B), Ib (E), II (H), and IIIa (K), followed by Alexa Fluor 594-conjugated donkey anti-rabbit IgG; while FimH was localized using biotinylated FimH/C complex, followed with FITC-conjugated streptavidin (A, D, G, J). Arrows mark the co-localization of surface-expressed uroplakins and FimH binding sties (A–L).

In bladder urothelial cells, uroplakins are expressed at the cell surface and interact with bacterial FimH during UPEC pathogenesis. We examined the expression of uroplakins in cultures of PD07i cells, an immortalized normal human urothelial cell line that is competent to undergo TNF-induced apoptosis [30],[31]. We sought to confirm that uroplakins are expressed in PD07i cells at the cell surface and that purified FimCH, recombinant FimH in complex with the FimC chaperone [32], interacts with surface-expressed uroplakin receptors. For this purpose we derived affinity-purified uroplakin antibodies that specifically recognize uroplakin proteins of human origin (Figure 1A, see Figure S1A for optimization of double staining immunofluorescence using these antisera). Under differentiation-inducing conditions that induce robust uroplakin expression [33], these antibodies detected punctate cell surface expression of human UP1a, UP1b, UPII and UPIIIa in PD07i cells (Figures 1B–1E). UPIa/UPII and UPIb/UPIIIa were shown to co-localize on the PDO7i apical cell surface (Figures 1F–1K). Following treatment of these cells with biotinylated FimCH (see Figure S1B) we observed distinct co-localization of FimCH with all the individual uroplakin subunits, suggesting that FimCH binds to uroplakin complexes containing all four uroplakins (Figure 2, panels A–L). These results indicate that PDO7i cells express all four major uroplakin subunits in complexes that are fully capable of interacting with FimH to mediate binding and potential downstream signaling events. The co-localization of FimCH to UPIIIa on the PDO7i cell surface suggests that FimH binding to the uroplakin receptor complex, which contains UPIIIa, could potentially lead to signaling events mediated by the UPIIIa cytoplasmic domain.

### Bacterial FimH induces UPIIIa phosphorylation and elevation in urothelial [Ca^2+^]_i_

**Figure 3:**
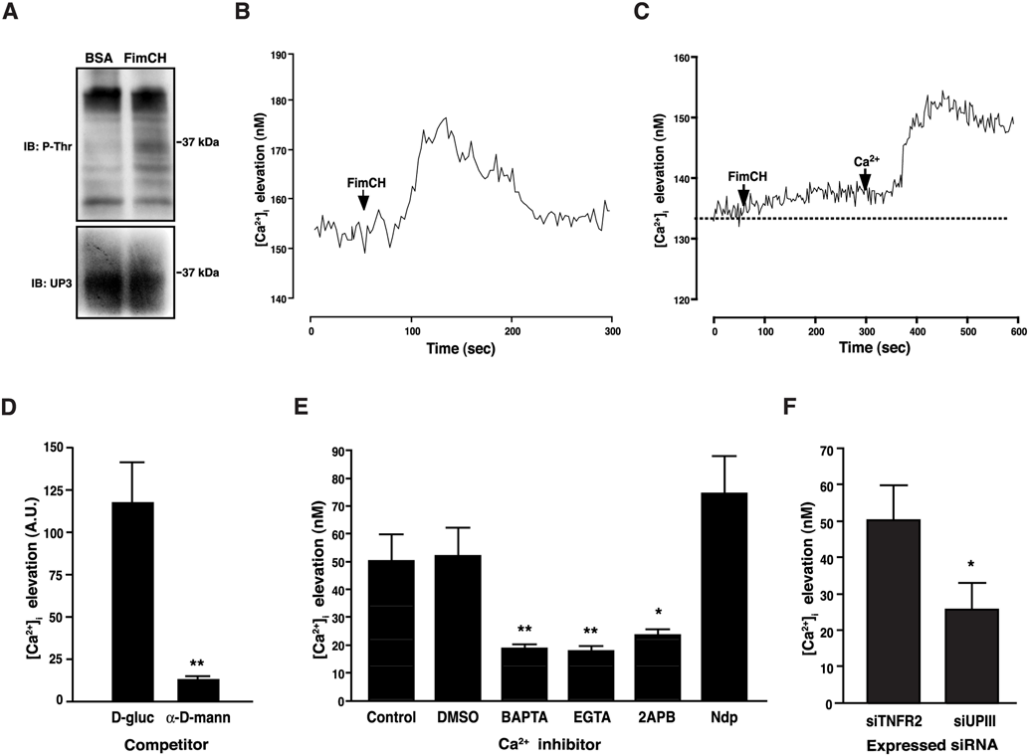
FimH induces UPIIIa phosphorylation and intracellular calcium elevation. (A) PD07i cells were stimulated with either 10 µg/ml FimCH or 10 µg/ml BSA for 30 minutes, and UPIIIa was immunoprecipitated from 100 µg cell extract with anti-UPIII antibody. Following electrophoresis and blotting, immunoprecipitated proteins were probed with an anti-phosphothreonine antibody (P-Thr). Blots were subsequently re-probed with an anti-UPIII antibody to determine total protein loading (UP3) (B) PD07i cultures were loaded with 5 µM Fura-2 and imaged for 5 minutes at 340 nm and 380 nm using real-time video fluorescence microscopy. FimCH (10 µg/ml) was added after establishing baseline Ca^2+^ concentrations collection for 1 min (arrow). The trace is the mean of a representative experiment of three replicates. (C) Extracellular Ca^2+^ is required for FimCH-induced urothelial Ca^2+^ elevation. PD07i cells were loaded with 5 µM Fura-2 and imaged for 10 minutes in nominal Ca^2+^-free medium. Cells were then exposed to 10 µg/ml FimCH, imaged for 5 minutes followed by addition of Ca^2+^ to a final concentration of 2.5 mM to the buffer solution and data were acquired for another 5 min. Baseline Ca^2+^ concentration is represented by the dotted line. The trace is the mean of a representative experiment of three replicates. (D) FimCH-induced [Ca^2+^]_i_ elevation is inhibited by pre-incubation with α-D-mannoside. FimCH was pre-incubated for 30 minutes with 25 mM α-D-mannoside or D-glucose. PDO7i cells loaded with 5 µM Fluo-4 AM were treated with 10 µg/ml of the pre-incubated FimCH. PDO7i cells were imaged at 488 nm using real-time video fluorescence microscopy and maximal fluorescence after FimCH treatment was subtracted from baseline. (E) Chelating intracellular or extracellular Ca^2+^ inhibits elevation of FimCH-induced [Ca^2+^]_i_. PD07i cells loaded with 5 µM Fura-2 were pre-incubated with 10 µM BAPTA-AM, 4 mM EGTA, 60 µM 2-APB, 10 µM nifidepine (Ndp), or equivalent amounts of DMSO for 30 minutes, followed by washing and exposure to 10 µg/ml FimCH. Maximal [Ca^2+^]_i_ was subtracted from baseline [Ca^2+^]_i_. Statistical significance is indicated at *p<0.05 or **p<0.001 and data are represented as mean±SEM. (F) Knockdown of UPIIIa expression inhibits FimCH-induced Ca^2+^ elevation. Control cells (PDO7isiTNFR2) or PD07isiUPIII cells were loaded with Fura-2 and imaged upon addition of 10 µg/ml FimCH followed by post-acquisition analysis. All experiments were repeated at least three times, and statistical significance is indicated at *p<0.05 and data are represented as mean±SEM.

Signal transduction cascades in many systems are associated with the phosphorylation of amino acid residues in the cytoplasmic domain of transmembrane receptors [34]. To determine whether UPIIIa functions as a signal transducer in urothelial cells, we examined UPIIIa phosphorylation upon FimCH binding in cultures of PD07i cells. PD07i cultures were treated with BSA as a control protein or with purified FimCH. After immunoprecipitation of cell lysates with an anti-UPIIIa antibody, immunoblots were probed with an anti-phosphothreonine antibody followed by an anti-UPIIIa antibody to determine loading (Figure 3A). Treatment with FimCH increased the level of phosphorylated UPIIIa protein relative to total UPIIIa. In contrast, probing of immunoblots with an anti-phosphotyrosine antibody did not reveal any UPIIIa phosphorylation in response to FimCH (data not shown). UPIIIa phosphorylation was also detected in response to exposure of PD07i cultures to UPEC strain NU14 followed by probing with an anti-phosphothreonine antibody (Figure S2). These results indicate that FimCH binding leads to UPIIIa threonine phosphorylation and are consistent with a possible role for UPIIIa as a transducer of UPEC-induced signals.

Intracellular calcium ([Ca^2+^]_i_) elevation is a common signaling mechanism downstream of many receptors. Since others and we have previously described [Ca^2+^]_i_ elevation during infection with UPEC in culture [11],[25], we examined whether FimCH binding contributes to an elevation of the intracellular calcium level in urothelial cells. FimCH induced a transient [Ca^2+^]_i_ increase (Figure 3B) within approximately 40 seconds of treatment of PD07i cultures. The FimCH-induced [Ca^2+^]_i_ increase was attenuated in minimal calcium medium, but the increase was largely restored by the addition of calcium to the assay medium (Figure 3C, compare with 3B). We next examined whether the [Ca^2+^]_i_ response was dependent on FimCH binding to mannosylated proteins on urothelial cells. Inhibition of FimCH-induced [Ca^2+^]_i_ elevation was achieved upon pre-incubation of FimCH with α-D-mannopyranoside but not D-glucose (Figure 3D), thereby establishing the specificity of the FimCH-induced effects. To identify whether the calcium mobilized by FimCH was derived from an intracellular or extracellular pool, FimCH-induced [Ca^2+^]_i_ increase was measured in the presence pharmacological inhibitors selective for specific [Ca^2+^] pools. Calcium elevation was significantly inhibited by BAPTA and EGTA, intra- and extracellular chelators of calcium, respectively, suggesting a role for both types of calcium stores in FimCH-induced calcium elevation (Figure 3E; P<0.01). An inhibitor of the inositol-triphosphate (IP3)-gated intracellular calcium channel, 2-aminoethoxydiphenyl borate (2-APB), also significantly inhibited the calcium transient (P<0.05), while the L-type calcium channel blocker nifidipine (Ndp) had no effect (Figure 3E). These results suggest that IP_3_-gated calcium stores contribute to FimCH-induced calcium elevation. Extracellular calcium also contributes to FimCH-induced transients, but the precise mechanism of calcium influx is unclear.

To examine the role of UPIIIa in mediating calcium elevation, we used RNA interference to create a urothelial cell line with reduced expression of UPIIIa mRNA and protein (PD07siUPIII) [35]. Under differentiation-inducing conditions that induce uroplakin expression, UPIIIa mRNA induction in PD07siUPIII cultures was reduced to less than 1% of the parental PD07i cultures [35]. PD07siUPIII cells exhibited significantly lower FimCH-induced calcium elevation relative to a control PD07i cell line that stably expresses siRNAs against a control protein (Figure 3F; P<0.05). These data suggest that UPIIIa is involved in mediating FimH-induced calcium elevation in urothelial cells. Taken together, these findings suggest that the events initiated upon FimCH binding of urothelial cells include signal transduction through UPIIIa by phosphorylation of UPIIIa threonine(s) and elevation of the intracellular second messenger calcium.

### FimCH-induced [Ca^2+^]_i_ elevation requires UPIIIa threonine 244

**Figure 4:**
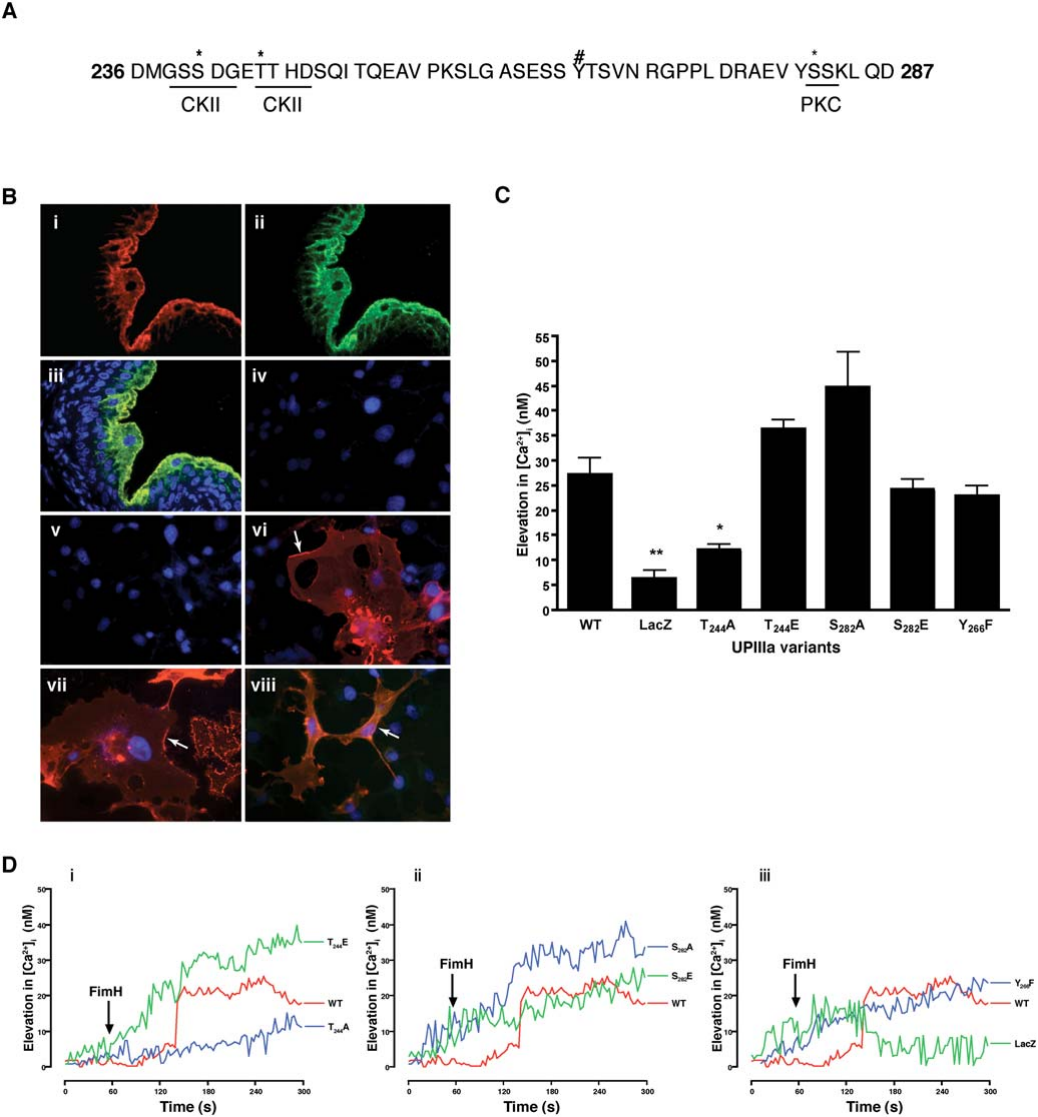
FimCH-induced Ca^2+^ elevation involves T_244_ mediated signaling. (A) The cytoplasmic tail of UPIIIa comprises of 52 amino acids with two predicted CK2 and one protein kinase C phosphorylation motif (underlined). T_244_ and S_282_ (indicated by asterisks) were mutated to A/E and Y_266_ (indicated by pound) to F to identify the phosphorylation site involved in FimCH-mediated Ca^2+^ signaling. (B) Mouse bladder sections were stained with P3 antibodies (red, i; see text) and AU1 antibody (green, ii), and the images were merged to confirm specificity (iii). COS-7 cells were infected with UPIb (iv) or UPIIIa adenovirus (v) and stained for UPIIIa protein with P3 antibodies. COS-7 cells co-infected with UPIb and UPIIIa viruses were stained with P3 antibodies (red, vi and vii) or P3 and AU1 (viii). Arrows indicate staining consistent with surface expression (vi–viii). Arrows indicate staining consistent with surface expression (vi–viii). (C) T_244_ mediates FimCH-induced Ca^2+^ elevation. Data acquired as in (D) were subject to post–acquisition analysis by subtracting maximal Ca^2+^ elevation following addition of 10 µg/ml FimCH from baseline Ca^2+^ concentration in COS-7 cultures. COS-7 cultures were previously infected with recombinant adenoviruses encoding UPIb and either wild type UPIIIa or UPIIIa variants or LacZ. At 24 h post-infection, traces of FimCH-induced Ca^2+^ elevation were acquired and reveal a requirement for T_244_ mediated signaling. (D) Representative traces are shown for T_244_ (i), S_282_(ii) and T_266_(iii) along with WT. All experiments were repeated three or more times, and statistical significance is indicated at *p<0.05 or **p<0.001 and data are represented as mean±SEM.

The UPIIIa cytoplasmic domain consists of ∼52 amino acids that harbor multiple potential phosphorylation sites (Figure 4A). We examined the involvement of threonine-244 (T_244_), and serine-282 (S_282_), residues predicted to be part of casein kinase II (CK2) and protein kinase C (PKC) phosphorylation motifs, in FimCH-induced calcium elevation. We also examined the involvement of tyrosine-266 (Y_266_), a potential phosphorylation site homologous to tyrosine-249 previously implicated in UPIII signaling in *Xenopus* oocytes. Specific mutations in a UPIIIa cDNA were generated that converted the potential phosphorylation sites to either glutamic acid or alanine, to mimic constitutive phosphorylation or block phosphorylation, respectively. Similarly, Y_266_ was mutated to phenylalanine to block phosphorylation. We utilized COS-7 cells for heterologous expression of the mutated UPIIIa variants since they lack endogenous UPIIIa expression [36]. Recombinant adenoviruses encoding the UPIIIa proteins were used to co-infect COS-7 cells along with an adenovirus encoding UPIb which, in culture, mainly harbor high mannose glycan [19],[23] that should therefore be able to serve as FimCH receptor. Immmunofluorescence studies were used to confirm heterologous surface uroplakin expression with recombinant adenoviruses. An affinity-purified antibody to UPIIIa, P3, was used in this experiment (see Figure S3 for specificity). Although infection of COS-7 cells with recombinant adenoviruses encoding either UPIIIa alone did not result in detectable uroplakin expression, co-infection of COS-7 cells with UPIb and UPIIIa viruses resulted in a significant surface UPIIIa expression detectable using either AU1 or P3 antibodies (Figure 4B iv–viii) [19],[23]. Like wild type UPIIIa, infection of COS-7 cells with recombinant adenoviruses encoding UPIIIa site-directed mutants also resulted in detectable UPIIIa surface staining when co-expressed with UPIb (Figure S4).

We next examined the ability of wild-type and mutated UPIIIa variants to mediate [Ca^2+^]_i_ elevation in response to FimCH. Following exposure to FimCH, cultures co-infected with wild type UPIIIa and UPIb viruses exhibited increased [Ca^2+^]_i_, whereas [Ca^2+^]_i_ elevation was not observed in cultures co-infected with UPIb and LacZ viruses, (Figures 4C and 4D). The kinetics of FimCH-induced [Ca^2+^]_i_ increase in COS-7 cells were slightly delayed and sustained compared to stimulation of PD07i cultures (compare Figures 4D and 3B), perhaps due to the expression of additional uroplakins in PD07i cultures or other urothelial-specific factors absent in the COS-7 heterologous expression system. Nonetheless, finding UPIIIa-associated, FimCH-induced [Ca^2+^]_i_ increases demonstrates the utility of COS-7 cells for uroplakin structure/function studies. Expression of the phosphorylation-deficient T_244_A UPIIIa variant was associated with a diminished FimCH-induced calcium elevation in COS-7 cells (Figure 4D-i, p<0.05), but the S_282_A and the Y_266_F variants retained responsiveness to FimCH (Figure 4D-ii and -iii), suggesting a specific requirement for T_244_ phosphorylation as a mediator UPIIIa signaling. The S_282_A variant (Figure 4D-ii) had an unexpected stimulatory effect on [Ca^2+^]_i_ elevation in response to FimCH while the constitutively phosphorylated T_244_E mutation reversed the inhibitory effect observed with the alanine replacement (Figure 4C and 4D). These findings suggest that FimCH-induced phosphorylation of UPIIIa on T_244_ is important for mediating [Ca^2+^]_i_ elevation in urothelial cells.

### UPIIIa cytoplasmic tail phosphorylation is mediated by casein kinase II

**Figure 5:**
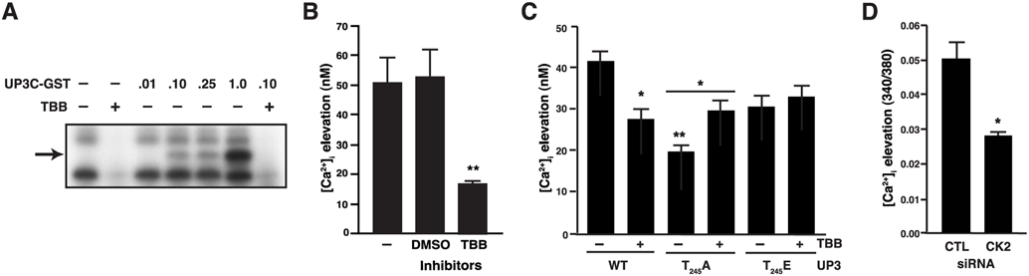
CK2 phosphorylates UPIIIa cytoplasmic domain and is required for FimCH-induced Ca^2+^ elevation. (A) UPIIIa cytoplasmic domain is phosphorylated by CK2 *in vitro*. Recombinant human CK2 (50 U) exhibited autophosphorylation (lane 1) that was blocked by 10 µM of the CK2 inhibitor TBB (lane 2). A fusion protein of the C-terminal domain of uroplakin IIIa with glutathione-S-transferase (UP3C-GST, arrow) served as a concentration-dependent substrate for phosphorylation by CK2 (lanes 3–6 containing 0.01 µg, 0.10 µg, 0.25 µg, or 1.00 µg UP3C-GST, respectively). CK2-mediated phosphorylation of 0.10 µg UP3C-GST was inhibited by TBB (lane 7). (B) Inhibition of CK2 abrogates FimCH-induced calcium elevation. Urothelial cells were loaded with Fura-2 AM and pretreated with 10 µM TBB (or equivalent concentration of vehicle (DMSO)) for 30 minutes, treated with FimCH and followed by imaging. Statistical significance is indicated at *p<0.05 and data are represented as mean±SEM. (C) COS-7 cells were infected with recombinant adenoviruses and FimCH-induced calcium was quantified as in (B) in the presence or absence of TBB. (D) RNA silencing of CK2 inhibits FimCH-induced calcium elevation in urothelial cells. PD07i cells transfected with siCK2 showed significantly reduced elevation in calcium as measured by the change in Fura-2 340/380 ratio compared to transfection with negative control siRNA. All experiments were repeated three or more times, and statistical significance is indicated by *p<0.05, **p<0.01.

Because T_244_ of the UPIIIa C-terminus resides within a predicted casein kinase II (CK2) phosphorylation motif, we examined the potential role of CK2 in UPIIIa signaling. We performed an *in vitro* kinase assay with recombinant CK2 and a fusion protein of the C-terminal 52 amino acids of UPIIIa with glutathione-S-transferase (UP3C-GST) as substrate. UP3C-GST underwent dose-dependent phosphorylation that was completely blocked by a pharmacological inhibitor of CK2, 4,5,6,7-tetrabromobenzotriazole (TBB), (Figure 5A). Since UP3C-GST serves as a CK2 substrate in cell-free system, we next examined the potential role of CK2 in FimCH-induced calcium elevation. While the vehicle had no effect on [Ca^2+^]_i_ elevation in PD07i cultures stimulated with FimCH, TBB significantly abrogated FimCH-induced [Ca^2+^]_i_ elevation (Figure 5B; P<0.01). To confirm the role of CK2 in modulation of FimH-induced signaling, we also examined the effects of TBB on uroplakin signaling in COS-7 cells expressing UPIIIa variants. TBB significantly reduced the [Ca^2+^]_i_ elevation induced by FimCH treatment of COS-7 cultures expressing wild type UPIIIa (co-transfected with UPIb; Figure 5C; P<0.05), and TBB significantly enhanced the calcium response in cultures expressing the T_244_A variant. To confirm the role for CK2 in FimH-induced calcium elevation, we utilized RNA interference to knockdown expression of CK2 in PDO7i cells. CK2 mRNA expression in PDO7i cultures transfected with CK2 siRNA was decreased by 75% compared with non-specific siRNA transfection (Figure S6, p<0.01). We then found that FimCH induced a significantly attenuated [Ca^2+^]_i_ elevation in CK2-silenced cells compared with the controls (Figure 5D). These results indicate that CK2 phosphorylates UPIIIa and mediates the FimCH-induced [Ca^2+^]_i_ elevation in urothelial cells.

### CK2 mediates urothelial invasion by UPEC invasion

**Figure 6:**
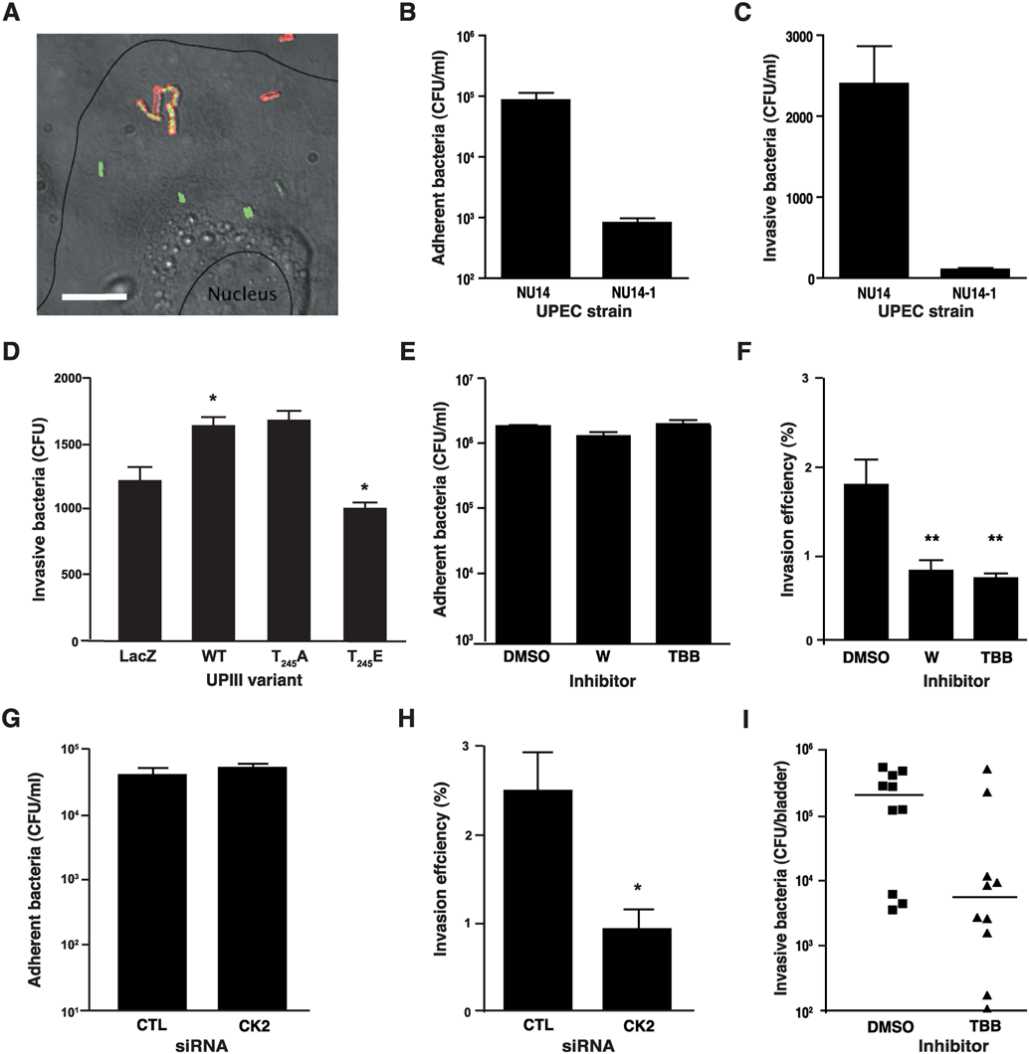
CK2 mediated FimH-dependent UPEC invasion *in vitro* and *in vivo*. (A) NU14 invades PD07i cells. PD07i cultures were infected with NU14-GFP (MOI 100; green) and stained for extracellular *E. coli* (red). (B) FimH mediates UPEC adherence to PD07i cells. Urothelial cells were infected with NU14 or NU14-1 at an MOI of 10, and infection proceeded for 2 h followed by washing. Cell lysates were plated on LB-agar to determine adherent bacteria. (C) FimH promotes UPEC invasion. PD07i cells were infected as above, washed and incubated in culture medium with 100 µg/ml gentamicin for 30 minutes followed cell lysis and plating onto agar. (D) NU14 invades 5637 cells infected with UPIIIa variants. Adenoviruses expressing various UPIIIa phosphorylation variants were used to infect 5637 cells followed by quantification of invasive bacteria. Inhibition of CK2 abrogates UPEC invasion but not adherence to urothelial cells (E&F). Adherence and invasion assays were performed as above with some modifications. Urothelial cells were infected with NU14 in the presence of 10 µM TBB, 1 µM wortmannin or vehicle control at an MOI of 100, medium was replaced after 1 hour with inhibitor-free media and infection was allowed to proceed for a further 1 hour followed by quantification of adherence and invasion as above. (G) Inhibition of CK2 using transfected siRNA does not affect adherence of NU14 to urothelial cells but reduces bacterial invasion of the bladder (H). *In vivo* TBB administration significantly reduced NU14 invasion of the bladder in female C57BL/6 mice (I). Mice were catheterized transurethrally with 10^8^ CFU of NU14 bacteria in PBS with TBB (10 µM) or a vehicle control (DMSO). Infection was allowed to proceed for 2 h followed by bladder removal and incubation in PBS with 100 µg/ml gentamicin *ex vivo* for 30 minutes at 37°C. Bladders were washed in antibiotic-free PBS and homogenized for quantification of bacterial colonization. With the exception of (I), all experiments were repeated three or more times, results are expressed as mean±SEM with statistical significance indicated at *p<0.05.

Previous reports demonstrate that UPEC strains invade urothelial cells *in vitro* and *in vivo*, and FimH is required for these processes [7],[13],[37]. We examined UPEC invasion of PD07i urothelial cells by the archetypal cystitis strain NU14 [38]. Intracellular NU14 were distinguishable from extracellular NU14 by fluorescence microscopy, confirming that PD07i cultures are useful for examining UPEC invasion of urothelial cells (Figure 6A). Consistent with previous reports, the FimH-deficient isogenic mutant of NU14-1 [38] was severely defective in both adherence and invasion of PD07i cells relative to wild-type NU14 (Figures 6B and 6C). To investigate the role of UPIIIa signaling in UPEC invasion, we utilized 5637 cells, a human carcinoma-derived urothelial cell line, to over-express wild-type and mutated UPIIIa. 5637 cells have been previously shown to support UPEC invasion through α3 and β1 integrins [39]. COS-7 was not utilized for these invasion studies because preliminary experiments showed that COS-7 cells supported high levels of basal NU14 invasion (data not shown). Since the 5637 cell line has constitutive expression of uroplakin proteins including the UPIIIa and its partner UPIb, adenoviral mediated overexpression of the wild-type or mutant UPIIIa alone was used to study their effects on UPEC invasion. Bacterial adherence to cells expressing the wild-type UPIIIa and the UPIIIa T_244_E variant were similar (data not shown). Wild type UPIIIa expression in 5637 cells significantly increased NU14 invasion relative to control cultures infected with control virus (Figure 6D; P<0.05). In contrast, expression of the UPIIIa T_244_E variant resulted in a much lower NU14 invasion than the wild type UPIIIa (P<0.05), consistent with a role for T_244_ in bacterial invasion.

To examine the role of CK2 in UPEC invasion, we quantified NU14 invasion in PD07i cultures in the presence of the CK2 inhibitor TBB. TBB had no effect on NU14 adherence to PDO7i cells (Figure 6E) but significantly decreased NU14 invasion (Figure 6F; P<0.01), yielding results comparable to treatment with the PI3-kinase inhibitor wortmannin, a known inhibitor of NU14 invasion [7]. To confirm the role of CK2 in NU14 invasion of urothelial cells, we used RNA interference to knockdown the expression of CK2. Knockdown of CK2 expression had no effect on the adherence of NU14 to PDO7i (Figure 6G) but significantly inhibited NU14 invasion when compared with a non-specific siRNA control (Figure 6H; p<0.05). These results indicate a role for CK2 in NU14 invasion of urothelial cells.

To determine the role of CK2 in UPEC invasion *in vivo*, we modified a murine UTI model [40]. Female C57BL/6 mice were instilled via transurethral catheter with 10 µl of NU14 bacterial suspensions at a concentration of 10^10^ CFU/ml plus either TBB or vehicle. After 2 hours, bladders were removed, opened, and incubated with gentamicin to kill extracellular bacteria before plating the tissue homogenates. Invasive NU14 levels were significantly reduced in the TBB-treated bladders relative to bladders of mice treated with vehicle alone (Figure 6I; P<0.05). These data suggest that CK2 is an important mediator of UPEC pathogenesis at the level of urothelial invasion.

### UPEC-induced apoptosis is mediated by UPIIIa signaling

**Figure 7:**
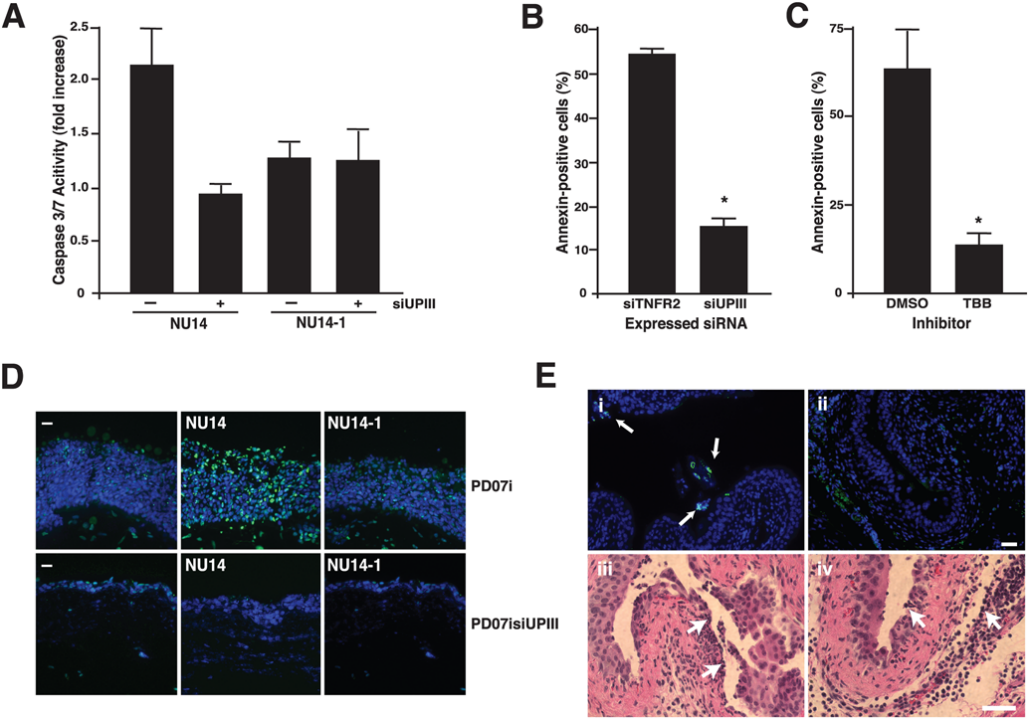
UPIIIa mediates FimH-induced urothelial apoptosis. FimH-induced caspase 3/7 activation requires UPIIIa (A). Caspase 3/7 was measured in culture extracts by cleavage of fluorogenic substrate. Induction of caspase 3/7 activity by NU14 (MOI 500) was significantly inhibited in PD07siUPIII cultures (*p<0.05) compared to control, and no induction of caspase 3/7 was detected in response to NU14-1. (B) PD07siTNFR2 cells or PD07siUPIII cells were treated with 10 µg/ml FimCH for 4 h. Apoptosis was visualized using fluorescent annexin V staining (green) and propidium iodide (red) staining. Annexin staining was quantified by manual counting of fluorescently labeled cells relative to brightfield images (see Methods). Annexin-positive cells were significantly reduced in siUPIII cells relative to siTNFR2 cells (*p<0.05*). (C) In experiments in the presence of inhibitors, PD07i cells treated with 10 µg/ml FimCH showed reduced annexin staining when treated with the CK2 inhibitor TBB compared to DMSO (*p<0.05*). (D) Organotypic raft cultures of PD07i or PD07siUPIII cells were incubated with NU14 or NU14-1 for 4 h at an MOI of 100. After processing, frozen sections were stained for TUNEL (green) and counterstained with DAPI (blue). Prominent TUNEL staining was induced by NU14 only in PD07i tissue. Images are representative of staining patterns obtained from TUNEL assay of four different raft cultures. (E) UPEC-induced apoptosis was also assessed in bladder sections using TUNEL staining (green). In *in vivo* experiments female C57BL/6 mice were infected with 10^8^ NU14 or NU14-1 for 6 hours in the presence of DMSO (i) or 10 µM TBB (ii). Bladder sections from DMSO treated animals exhibited foci of apoptosis (arrows) that were reduced or absent in TBB-treated animals. In H&E stained bladder sections from DMSO (iii) and TBB (iv) treated mice, infiltration of large numbers of inflammatory cells (white arrows) were observed in response to infection with 10^8^ NU14 for 2 hours, suggesting an intact innate immune response. Images are of representative sections and scale bars represent 50 µm. All experiments were repeated two or more times, and statistical significance is indicated at *p<0.05 and data are represented as mean±SEM.

Since FimH is known to induce urothelial cell death [11],[12], we examined the potential involvement of UPIIIa signaling in apoptosis. We first confirmed the role of FimH by demonstrating that NU14 induces a higher level of caspase activation, than the FimH-deficient strain NU14-1, in a PD07i cell line expressing a luciferase control plasmid [30] (Figure 7A; P<0.05). We next examined the role of UPIIIa by examining caspase activation in PD07siUPIII, an UPIIIa-deficient cell line. PD07siUPIII showed diminished caspase activation in response to NU14 when compared with control PD07i cultures, thereby confirming our previous observation that FimH-mediated urothelial cell death correlates with UPIIIa expression [35]. We also examined annexin V staining as an early marker of urothelial apoptosis [12],[30]. We found that annexin V staining was significantly more prevalent in control cultures than PD07siUPIII cultures (Figure 7B; P<0.05). Similarly, parent PD07i cultures treated with FimCH in the presence of the CK2 inhibitor TBB exhibited reduced annexin V staining, whereas FimCH treatment in the presence of vehicle were stained in significantly larger numbers (Figure 7C). We also examined FimCH-induced apoptosis following bacterial infection of organotypic raft cultures that recapitulate the urothelial differentiation program [31]. Organotypic cultures of PD07i cells exhibited FimH-dependent apoptosis, as indicated by TUNEL staining in NU14-treated cultures that was absent in NU14-1-treated cultures (Figure 7D). However, PD07siUPIII organotypic cultures did not undergo FimH-dependent apoptosis. Together, these data suggest that FimH-induced urothelial apoptosis is mediated by both UPIIIa and CK2 signaling.

We next examined the role of CK2 in UPEC-induced urothelial apoptosis *in vivo* by infecting mice with NU14 in the presence or absence of TBB and then detecting apoptosis by TUNEL staining [13]. TUNEL staining of bladder sections revealed foci of cells in the superficial layer of the bladder of control animals instilled with an NU14 suspension, but this was inhibited by TBB (Figure 7E, panels i and ii).

To determine whether UPIIIa signaling affected urothelial responses other than those involved in bacterial invasion and apoptosis, we examined TLR-dependent urothelial inflammatory responses *in vitro* following UPEC infection [41]. UPEC infection as well as treatment with the pro-inflammatory cytokine IL-1β induced similar level of CXCL-8 secretion in both PD07siTNFR2 and PD07siUPIIIa cultures (Figure S5). These results suggest that UPIIIa signaling is not required for urothelial inflammatory responses. In addition, *in vivo* murine UTI infection with NU14 elicited similar levels of bladder neutrophil influx in mice instilled with vehicle or the CK2 inhibitor TBB (Figure 7E, panels iii and iv). Taken together, our data suggest that UPIIIa and CK2 specifically mediate two key events in UTI pathogenesis, UPEC invasion of urothelial cells and urothelial apoptosis.

## Discussion

Uroplakins are critical components of the AUM that provide the bladder permeability barrier, yet AUM dynamics suggest the ability to modulate urothelial cell signaling [42]. The discovery of bovine UPIa as the receptor for FimH provided the first evidence for involvement of uroplakins in UTI pathogenesis [20],[22]. These interactions are presumed to mediate two key UPEC-induced events observed in cell culture and murine UTI models, namely rapid urothelial apoptosis and invasion of urothelial cells by UPEC [7],[11],[12]. While both events require activation of cell signaling cascades, the proximal mediator of these cascades has not been identified. This study provides evidence that UPIIIa is a membrane-proximal signal transducer of UPEC pathogenesis in the bladder.

### Uroplakins as functional UPEC receptors

Using monospecific antibodies for individual uroplakins, we showed that UPIa and UPIb co-localized with UPII and UPIIIa, respectively, in cultured human PD07i urothelial cells (Figure 1). This result is consistent with our previous finding that individual uroplakins preferentially form UPIa/UPII and UPIb/UPIIIa heterodimers, and this step is a prerequisite for uroplakin complex export from the endoplasmic reticulum [23],[43],[44]. Uroplakins residing outside the afore-mentioned, preferred heterodimers were much less colocalized (data not shown). The data reported here are also consistent with our previous reports: uroplakins in cultured bovine urothelial cells remain as heterodimers, whereas in normal urothelium *in vivo* these uroplakin heterodimers proceed to form heterotetramers, six of which then form the hexagonal 16-nm particle [43]. The fact that uroplakin staining appeared punctate suggests that uroplakins accumulated preferentially on apical microvilli of PD07i cells (Figures 1 and 2), similar to our previous observations in cultured bovine urothelial cells [45]. Taken together, our results establish that human urothelial PD07i cells express all four major uroplakins primarily as heterodimers.

Previous studies indicate that UPIa is the main UPEC receptor in normal urothelium, yet data presented here suggest that UPIb may serve as an alternative FimH receptor in cultured urothelial cells (Figure 2). Of the four major uroplakins present in mouse urothelial plaques, we previously showed that UPIa is the only uroplakin bearing large amounts of high mannose glycan [21]. Consistent with this result, type 1-piliated *E. coli* or recombinant FimCH preferentially bound UPIa, and similar results were obtained with human uroplakins, suggesting that UPIa is the main UPEC receptor *in vivo* [20],[21],[22]. UPIb and UPIIIa harbor mainly complex sugars [17],[18],[21]. UPII is unlikely to function as a UPEC receptor, for proteolytic processing of pro-UPII that harbors three N-glycosylation sites eliminates those sites from mature UPII [46]. Somewhat unexpectedly, we found here that FimCH co-localized with all four uroplakins (Figure 2), suggesting that FimH may interact with either uroplakin Ia/II or Ib/IIIa heterodimers on PD07i cells. However, this is consistent with previous observations that heterologous expression of UPIa/UPII and UPIb/UPIIIa pairs in COS-1 cells resulted in both UPIa and UPIb bearing high mannose glycans [43]. Together these findings suggest that uroplakins exist mainly as heterodimers in culture models, and both UPIa/UPII and UPIb/UPIIIa pairs are functional FimH receptors. Therefore, while culture models are convenient for identifying molecular mechanisms of FimH-induced UPIIIa signals, we also validate these findings *in vivo*, where UPIa is the major FimH-receptor (Figures 6 and 7).

### FimH-induced uroplakin III signaling

Sperm-egg fusion in *Xenopus* provided the first evidence for a signaling role for uroplakins with phosphorylation of the cytoplasmic tail of xUPIII on Y_249_ [27]. In contrast to these results, our data implicate phosphorylation of T_244_ as a key early event induced by FimH binding in culture. Indeed, we found that mutation of hUPIIIa Y_266_, the homologous position to xUPIII Y_249_, did not influence FimH-induced calcium elevation, suggesting that signaling initiated by modification of hUPIIIa Y_266_ is not involved in urothelial responses to UPEC. This difference in phosphorylation site utilization may represent ligand-specific, species-specific, and/or tissue-specific signaling. We do not however rule out the possibility that yet-unidentified physiological ligands induce human UPIIIa phosphorylation on tyrosine residues using conserved mechanisms comparable to *Xenopus*. On the contrary, we speculate that the presence of multiple putative phosphorylation sites on the cytoplasmic tail of UPIIIa mediate responses to diverse stimuli and involvement in multiple biologic processes, such as fusiform vesicle exocytosis that expands the AUM surface area during bladder filling [47].

Our findings that UPIb was sufficient for UPIIIa-mediated responses induced by FimH in COS-7 cells (Figures 4 and 5) is consistent with the conserved function of complexes containing tetraspanin proteins. Tetraspanins typically function as transmembrane linkers in a complex with other proteins that then transduce signals [48]. Thus in the case of uroplakin complexes, tetraspanins function as the ligand binding module (UPIa/UPIb, and UPIII tranduces FimH-induced signals. The precise mechanism by which FimH-uroplakin interactions transduce signals across the highly impermeable urothelial apical membrane remains unknown. However, Kong and colleagues have recently used cryo-electron microscopy to show that FimH binding induces gross conformational changes in the entire uroplakin receptor complex, including movement of the transmembrane helices to potentially induce structural changes in the uroplakin cytoplasmic tails (Xiang-Peng Kong, New York University School of Medicine, personal communication). These independent results bolster our hypothesis that FimH-induced changes in the transmembrane helices of the uroplakin receptor complex mediate the transmembrane signal that triggers bacterial invasion and host cell responses.

### Uroplakin III signaling in UTI pathogenesis

The finding that UPIIIa signaling initiates [Ca^2+^]_i_ elevation has important implications for UPEC pathogenesis. FimH-mediated calcium elevation occurs as a result of calcium release from intracellular stores and by influx from extracellular sources, and calcium elevation promotes global responses critical to UPEC pathogenesis including cytokine stimulation, membrane trafficking and apoptosis [25],[49]. The findings here extend our earlier observations that sustained calcium elevation is associated with UPEC-induced apoptosis [11] and suggest a role for calcium signaling in both immediate early host responses and subsequent events. Our finding that UPIIIa initiates urothelial calcium also presents a testable hypothesis for the mechanism underlying the modulation of calcium-dependent exocytosis of fusiform vesicles during bladder stretch [47].

Approximately 25% of UTI patients suffer recurrent infections, and invasion of urothelial cells in culture and in mice promotes the establishment of a drug-resistant UPEC niche that may underlie recurrent UTIs in humans [37]. The factors required for UPEC invasion *in vitro* include expression of type-1 pili, intracellular signaling events mediated by PI3-kinase, and activation of Rho-GTPase [50]. In this study, we examined the role of the UPIIIa signaling in UPEC invasion *in vitro* and in mice utilizing the CK2 inhibitor TBB, because mutagenesis studies implicated a putative CK2 site at T_244_ of the UPIIIa cytoplasmic tail. TBB inhibition of UPEC invasion suggests that FimH-mediated mechanisms of invasion in the bladder also rely on UPIIIa and CK2. The UPIIIa variant T_244_E, designed to mimic constitutively phosphorylated UPIIIa, inhibited invasion, while the T_244_A has no such effect. Thus, the phosphorylation status of T_244_ has implications for UPEC invasion. The absence of correlation between calcium signaling with the T_244_ mutants and UPEC invasion may result from distinct kinetics. Calcium signaling is an early event occurring within minutes while invasion is measured within hours. These results suggest that UPIIIa-induced signals may interact with other signals induced by UPEC binding to modulate UPEC invasion of urothelial cells. For example, recent studies demonstrate that UPEC adherence induces cAMP increases via TLR4 signaling, and this increase hinders UPEC invasion [51]. The salutary effects of TBB on UPEC invasion *in vivo* suggest CK2 is a novel therapeutic target for intervention in UTI at the level of inhibiting UPEC invasion.

It has been postulated that urothelial apoptosis is a host defense mechanism against UPEC insult, because inhibiting apoptosis in a murine UTI model was detrimental to bacterial clearance from the bladder [13]. We previously showed that UPEC-induced apoptosis is mediated by type 1 pili and occurs through activation of intrinsic and extrinsic cell death pathways [11],[12]. In this study we demonstrate that FimH-induced apoptosis is dependent upon the expression of UPIIIa and is inhibited by abrogation of signals downstream of UPIIIa. We have also recently shown that increasing levels of UPIII expression in urothelial cells renders cells more susceptible to FimCH-dependent apoptosis [35]. Interestingly, UPIIIa expression or signaling does not appear to be required for urothelial inflammatory responses, suggesting that UPEC-induced signals emanating from UPIIIa are separate and unique from simultaneous inflammatory signals mediated by TLRs. The potential interactions between simultaneous UPEC activation of both pro-apoptotic and pro-inflammatory pathways is intriguing but uncharacterized.

**Figure 8:**
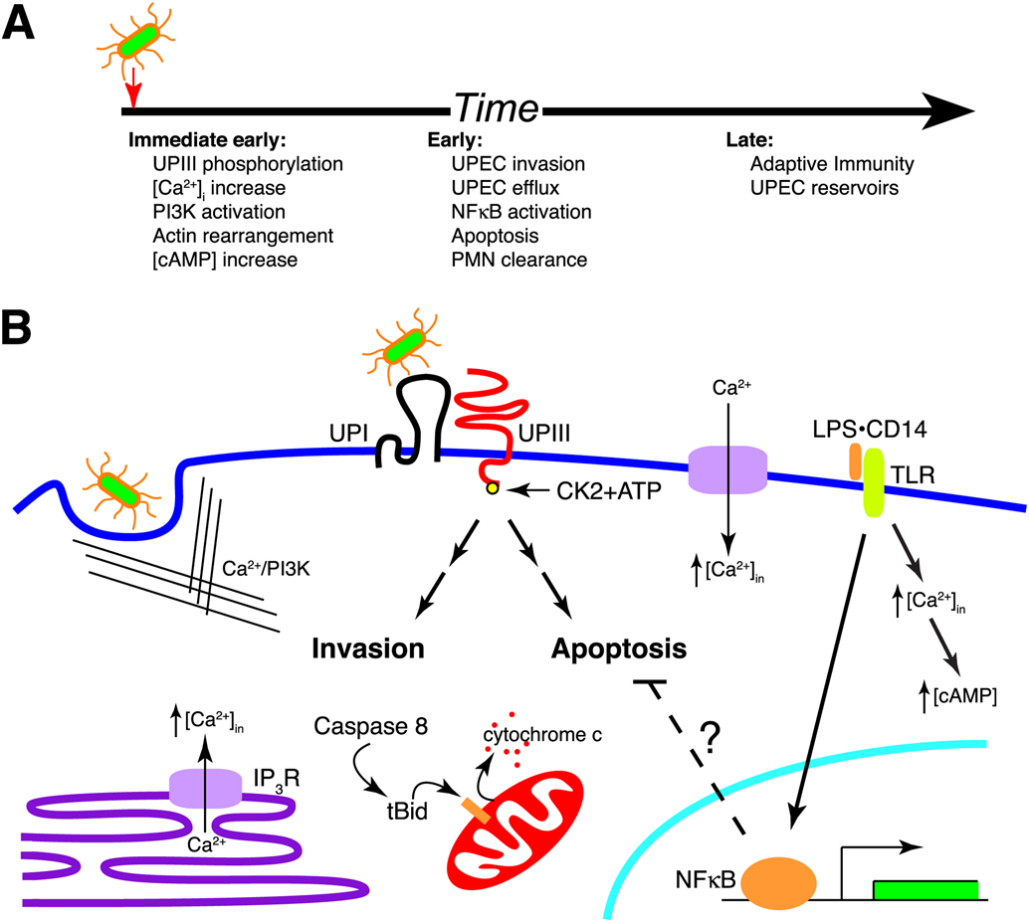
Model of UPIIIa mechanisms of UPEC pathogenesis. (A) UTI pathogenesis in the bladder occurs in distinct kinetic phases. Immediate early host responses include UPIIIa phosphorylation and increased [Ca^2+^]_i_ (this study), PI3K activation [7], actin rearrangement [50], and increased cAMP [51]. Subsequent early host responses include UPEC internalization [7], NFκB-dependent chemokine production and modulation [12], urothelial apoptotic cascades [11], and bacterial clearance mediated by neutrophils [10]. Late events include adaptive responses that confer protective immunity [40] and establishment of stable UPEC reservoirs within the urothelium [37]. (B) UPEC interaction with uroplakins leads to phosphorylation of the UPIIIa cytoplasmic tail by CK2 and the initiation of two signaling cascades. Elevation in intracellular calcium from intra- and extracellular stores and the associated recruitment of other signaling molecules activates host cell cytoskeletal elements and the endocytosis of UPEC. UPIIIa phosphorylation activates an unknown signaling intermediate that initiates intrinsic and extrinsic apoptotic cascades. Pro-survival signals initiated by TLR activation may shift the balance away from UPIIIa-induced pro-apoptotic signals in those cells where UPEC successfully establish stable intracellular populations.

We propose a model of UPEC pathogenesis that combines the known responses to UPEC with the UPIIIa signaling described in this study (Figure 8). The host response is characterized by events in distinct kinetic classes representing immediate early signaling responses, early innate responses, and culminating in late events. Our findings identify FimH-induced UPIIIa signaling mediated by CK2 and subsequent [Ca^2+^]_i_ elevation as the immediate host responses. These membrane-proximal signals activate two seemingly opposed urothelial outcomes – urothelial invasion by UPEC and UPEC-induced apoptosis. Bacterial invasion occurs through activation of the host cytoskeleton and utilization of conserved endocytic pathways. The demonstration of a role for the endocytic/exocytic machinery of urothelial fusiform vesicles in UPEC invasion leads us to speculate that UPEC-mediated UPIIIa signaling is a bacterial pathogenesis mechanism initiated to activate the endocytic machinery in urothelial cells, thereby gaining access to an intracellular sanctuary [9]. Apoptosis, triggered by an as-yet-unknown signaling intermediate, rids the host of infection and may be considered part of a robust innate response. In the event of a successful innate response, the urothelial cell undergoes apoptosis and is eliminated in the urine. We postulate that initiation of apoptosis may require a threshold of UPIIIa signaling that varies from cell-to-cell depending on levels of UPIIIa expression or expression of other cellular factors. This scenario is consistent with heterogeneous bladder lesions induced by UPEC and the recent finding that urothelial cells may derive from multiple progenitor populations [52]. In the absence of this robust clearance mechanism, invasive bacteria are likely to establish stable reservoirs. Successful establishment of this immune-resistant niche may also require pro-survival TLR signals that shift the equilibrium away from apoptosis and promote cell survival. Irrespective of the precise mechanisms, the balance between pro-survival and pro-apoptotic mechanisms may determine UPEC pathogenesis in the urinary bladder.

In summary, two critical pathogenic results of UPEC-urothelial interactions, bacterial invasion and host cell apoptosis, involve UPIIIa and are associated with CK2 and calcium signaling. This first description of human UPIIIa as a signal transducer raises important questions regarding the normal physiological function of this highly expressed protein in the mammalian urinary bladder. Determining how UPIIIa-mediated signals interact with the recently characterized role of TLR4-induced cAMP modulation will illuminate key aspects of both urothelial biology and host-pathogen interactions in UTIs. Finally, identifying calcium and CK2 as immediate early host responses to UPEC offers novel therapeutic targets for intervention in UTIs.

## Methods

### Mice

Female C57BL/6 mice were obtained as specific-pathogen-free from Jackson Laboratories (Bar Harbor) and housed in Northwestern University's Center for Comparative Medicine. After a 1-week acclimatization, 6- to 10-week old mice were anesthetized with isoflurane and inoculated by transurethral catheter with 10^8^ CFU *E coli* in 10 µl to minimize reflux to the kidneys [53]. All experiments were conducted using protocols approved by the Animal Care and Use Committee of Northwestern.

### Bacterial strains

NU14 was obtained from the urine of a cystitis patient, and NU14-1 lacks functional type 1 pili [38]. Bacteria were cultured in static Luria broth at 37°C to promote type 1 pilus expression [54] that was confirmed by mannose-sensitive haemagglutination [55],[56]. For *in vitro* infections, bacteria were centrifuged and washed once in cold PBS. Bacteria were resuspended in culture medium at the appropriate multiplicity of infection (MOI). Bacterial preparation for *in vivo* studies was performed as previously described [40]. FimCH, a stabilized form of FimH in complex with the FimC chaperonin, was a kind gift from Dr. Hultgren.

### Biotinylation of FimH/C complex

Briefly, 1 mg of FimH/C in 1 ml 50 mM Na_2_CO_3_-NaHCO_3_ (pH 9.0) was mixed with 50 ml sulfo-NHS-biotin (1 mg/ml; Cat# 21217, Pierce Chemical, Rockford, IL) freshly dissolved in water. The mixture was left on ice for 2 hours, and the buffer was changed to Tris-buffered saline (TBS) (150 mM NaCl, 50 mM Tris-HCl, pH 7.5) through ultrafiltration (Centricon, 10 k-Da cutoff, Millipore, Bedford, MA) to remove the free biotin [22].

### Cell culture

PD07i cells are an immortalized human urothelial cell line previously established by infection of normal human urothelial cells (obtained by dissociation of pediatric bladder) with a retrovirus encoding E6E7 of HPV type 16 [30]. PD07i cells were maintained in EpiLife medium (Invitrogen). PD07i cells were used to establish a stable cell line in which UPIIIa expression was blocked using short hairpin RNA (shRNA) specific for UPIIIa and designated as PD07isiUPIII (Clone V2HS_95009, OpenBiosystems). As a control for the retroviral vector used in these studies, PD07i cells expressing shRNA targeting TNF receptor 2 designated PD07isiTNFR2 were also employed to demonstrate specificity (clone V2HS_94072, OpenBiosystems) [30]. Silencing of UPIIIa gene expression was confirmed by real-time analysis, and expression was reduced approximately 99% (Thumbikat et al., manuscript in review). COS-7 cells and 5637 cells were used for heterologous expression of uroplakins and were cultured in DMEM containing 10% fetal bovine serum. Urothelial biomimetics cultures were generated by organotypic raft culture as previously described [31].

### Culturing of PD07i cells for immunofluorescence

PDO7i cells were suspended (1×10^5^ cells/ml) in a complete bladder urothelium culture medium (FAD medium): 1∶1 mixture of DME and Ham's FI2 medium, containing 10% FCS, hydrocortisone (0.5 mg/ml), cholera toxin (5 ng/ml), insulin (5 ug/ml), epidermal growth factor (15 ng/ml) [33]. 2×10^5^ cells were seeded into upper chamber of a 24-mm Transwell with 3 ml of FAD medium in the lower chamber. Culture medium was changed regularly every 3 days (3 ml in the basolateral chamber and 2 ml in the apical chamber). Cells were fixed at post-confluence day-5 with fresh prepared 4% paraformaldehyde in PBS pH 7.4 at 25°C for 15 min before immunostaining.

### Uroplakin immunostaining

For single uroplakin immunostaining, cells were fixed and incubated at 4°C overnight with anti-UPIa, Ib, II, and IIIa antisera 1∶200 diluted in 1% fish skin gelatin in PBS (pH 7.4). The primary antibodies were detected with an Alexa Fluor 488- conjugated donkey anti—-rabbit IgG (Cat# A-21206, Invitrogen, Carlsbad, CA) in PBS (pH 7.4) with 1% FSG fish skin gelatin. Cells were also incubated with 1 ug/ml propidium iodide (Cat # 537059, Calbiochem, San Diego, CA) at 25°C for 5 min for nuclear staining. For uroplakin double staining, monovalent Fab fragment of rhodamine-conjugated donkey anti-rabbit IgG (Cat# 711-297-003, Jackson Immunoresearch lab, West Grove, PA) was used for detecting and blocking of double labeling of rabbit anti-UPIa, and Ib antisera according to http://www.jacksonimmuno.com/technical/fab-blok.asp. Fixed cells were first stained with rabbit anti-uroplakins Ia, or Ib antisera, respectively, followed by detecting and blocking with a monovalent Fab fragment of rhodamine-conjugated donkey anti-rabbit IgG, then the samples were briefly fixed for 15 min with 4% paraformaldehyde in PBS (pH 7.4), and after 15 min neutralized using 100 mM NH_4_Cl in PBS (pH 7.4), samples were double stained with anti-UPII or UPIIIa rabbit antisera, and finally detected using the Alexa Fluor 488 conjugated donkey anti-rabbit IgG.

### Uroplakins and FimH double staining

Fixed cells were incubated for 1 hr at 25°C in a TBS+ buffer (150 mM NaCl, 1 mM CaCl_2_, 1 mM MgCl_2_, 50 mM Tris-Cl pH 7.4) with 1% fish skin gelatin, 10 ug/ml of biotinylated FimH/C, and rabbit anti-uroplakin Ia, Ib, II, and IIIa antisera (1∶200 dilution). The biotinylated FimH/C and anti-uroplakins antibodies were detected using FITC-conjugated streptavidin and Alexa Fluor 594-conjugated donkey anti-rabbit IgG (Cat# A21207, Invitrogen, Carlsbad, CA), respectively.

### Uroplakin immunoblotting

0.1 ug of bovine or human AUM proteins or 10 ng of biotinylated FimH/C were resolved electrophoretically by 17% SDS-PAGE and transferred onto a nitrocellulose membrane (Cat# 162-0112, Bio-Rad, Hercules, CA). The individual uroplakins were incubate with rabbit antisera against UPIa, Ib, II, and IIIa, and followed by of 1 mg/ml horseradish peroxidase (HRP)-labeled goat anti-rabbit IgG (Cat# A8275, Sigma, St. Louis, MO). The biotinylated FimH/C was detected directly by HRP-labeled streptavidin (Cat# S5512, Sigma, St. Louis, MO).

### Detection of UPIIIa phosphorylation

PDO7i cells (5×10^6^) were incubated with 10 µg/ml FimCH for 15-30 minutes in culture medium followed by lysis in modified RIPA buffer. Lysates were precleared with protein A sepharose beads (Santa Cruz Biotechnology) for 90 minutes, followed by addition of anti-human UPIIIa polyclonal antibody (rabbit antibody raised against the human UPIIIa peptide QTLWSDPIRTNQL; Invitrogen) and immunoprecipitation of the complex with protein A sepharose. Eluted proteins were fractionated on 4–15% SDS-polyacrylamide gels and transferred to Immobilon-P membrane (Millipore). Blots were probed with anti-phosphotyrosine or anti-phosphothreonine antibodies (Cell Signaling Technologies, P-Tyr-100 monoclonal and P-Thr-polyclonal respectively) and imaged by chemiluminescence.

### Detection of intracellular calcium

Intracellular calcium ([Ca^2+^]_i_) was quantified in fura-2/AM-loaded PDO7i or COS 7 cells exposed to 10 µg/ml FimCH by video fluorescence imaging [57]. Briefly, cells grown on chambered coverslips were rinsed in modified Hank's balanced salts (HBSS) and incubated for 30 minutes in 5 µM fura-2/AM. The cells were then washed in modified HBSS and monitored at 520 nm after excitation at 340 nm (bound Ca^2+^) and 380 nm (free Ca^2+^) using a 20× water immersion lens. Fluorescence was analyzed from at least 30 cells in each experiment using MetaFluor software (Universal Imaging Corporation) from regions of interest after correction for system background, shading errors, and autofluorescence of unloaded cells. [Ca^2+^]_i_ was calculated by the ratio method [58]. Inhibitors 2-aminoethoxydiphenyl borate, nifidipine and CK2 inhibitor 1 (TBB) were from Calbiochem, and wortmannin, EGTA and BAPTA-AM were from Sigma. For inhibitor experiments, cells were pre-incubated with drugs or vehicle for 30 minutes at 25°C prior to FimCH exposure. [Ca^2+^]_i_ elevation was calculated by subtracting the baseline [Ca^2+^]_i_ from the maximal calcium value.

### Site-directed mutagenesis and expression of uroplakins

The human UPIb and UPIIIa cDNA clones were kind gifts from Dr. Jennifer Southgate. Individual uroplakin cDNAs were cloned into the multiple-cloning site of pDONR followed by transfer into the pAd/CMV/V5-DEST adenoviral vector with Gateway technology (Invitrogen). The resulting plasmids were linearized by digestion with *Pme*I and transfected into HEK 293 cells. Recombinant adenoviruses were titered and stored at −70°C until use. UPIIIa variant adenoviruses were generated (T_244_A, T_244_E, S_282_A, S_282_E, and Y_266_F) by site-directed mutagenesis of the wild type pAd/CMV/UP3 sequence. COS-7 cells and 5637 cells were infected with uroplakin adenoviruses for 4 h (MOI = 100) and incubated for 20 hours before experiments.

### Affinity purification of UPIIIa antibodies

Anti-AUM antiserum was affinity purified to yield a UPIII-specific “P3” fraction as previously described [59]. Briefly, UPIIIa peptide (S87 to K101) was immobilized on nitrocellulose. Following blocking of the filter with BSA, the filter was incubated with anti-AUM for 1 h at room temperature, and bound antibodies were then eluted with 50 mM diethylamine (pH 11.5) and immediately neutralized with 1M Tris-HCl (pH 7.4).

### *In vitro* phosphorylation of UPIIIa C-terminus

The DNA sequence encoding the C-terminal 52 amino acids of UP3 (UP3C) was cloned into the Sgf I and Pme I sites of the glutathione-S-transferase (GST) vector pFN2A (Promega). UP3C-GST fusion protein was purified from *E. coli* strain BL21 using standard techniques. Casein kinase II-mediated phosphorylation of the C-terminal domain of uroplakin IIIa was performed using standard protocols [60]. Briefly, kinase reactions were performed in 20 mM Tris-HCl pH7.5, 150 mM KCl, 5 mM MgCl2, and 500 µM DTT containing 20 µCi ^32^P-ATP (3000 mCi/mmol), cold ATP to 5 µM, and 50 U recombinant human recombinant CK2 (Calbiochem 218701). Some reactions also included 10 µM CK2 inhibitor TBB (Calbiochem 21708). Reactions were incubated at 30°C for 10 min before termination with SDS loading dye and electrophoresis through 10% polyacrylamide.

### RNA interference of Casein Kinase 2

PD07i cells were transfected overnight using Lipofectamine 2000 (Invitrogen) with a total of 200 pmol/well (6-well plates) or 40 pmol/well (24-well plates and chambered coverslips) of combination of equal amounts of each dsRNA duplex from the CSNK2A1 Validated Stealth™ RNAi Duopak (Invitrogen) or the equivalent amount of Stealth™ RNAi Negative Control Med GC duplex as a negative control. The following day, the transfection media was removed and fresh culture media was added to the cells. Forty-eight hours following transfection, the cells were used to determine intracellular invasion efficiency, caspase 3/7 activation following infection with NU14 or [Ca^2+^]_I_ elevation following FimCH treatment.

### Bacterial adherence and invasion

PD07i cells were infected with NU14 (MOI 10) and centrifuged twice at 600×g for 2.5 min. Infected cultures were incubated at 37°C for 2 h. To measure adherence, cells were washed 4 times with PBS and incubated with 0.05% trypsin/0.1%Triton X-100 for 10 minutes to lyse cells. Lysates were harvested, plated on LB-agar containing appropriate selection, and colonies were counted to quantify bound bacteria. To measure invasion, cells were infected and washed as above before incubating in 100 µg/ml gentamicin for 30 minutes at 37°C and plating lysates as described above. For inhibitors, cells were infected (MOI 100) in the presence of inhibitor or vehicle and incubated at 37°C 1 h and then changing to inhibitor-free media for 1 h. Mice were infected via transurethral catheter with 10 µl of bacterial suspension containing 10^8^ CFU [40] containing 10 µM TBB or vehicle. After 2 h, animals were sacrificed and opened bladders were incubated in PBS containing 100 µg/ml gentamicin for 30 minutes at 37°C before rinsing and plating tissue homogenates.

### Fluorescence microscopy for UPEC detection

To detect intracellular UPEC, PD07i cells were seeded into 4-well chambered slides and grown to confluence. Cells were infected at an MOI 100 with NU14/pcomGFP [61] and incubated at 37°C 2 h. To stain extracellular bacteria, cells were incubated with biotinylated anti-*E. coli* antibody (Abcam) in Epilife/1% BSA at 37°C. Cells were washed and fixed in 1% PFA then blocked in PBS/1% BSA before incubating with Streptavidin-AlexaFluor 594 (Invitrogen). Each step occurred for 30 minutes at 25°C and slides were washed three times with PBS between each step. Images were acquired with OpenLab (Improvision) using a 63× HCX PlanApo objective on a Leica DM-IRE2 microscope outfitted with an OCRA2 camera (Hamamatsu), and fluorescent channels were merged with DIC imaging; curves demarking the nucleus and cell margins were added to aid interpretation.

For detection of uroplakin expression, COS-7 cells were infected with recombinant adenoviruses encoding human uroplakins (MOI 100). The following day, cells were fixed for 15 min. at 25°C with freshly prepared 4% paraformaldehyde in PBS pH 7.4. Cells were then rinsed and quenched with 10 mM glycine in PBS pH 7.4 at 25°C for 10 min. The cells were then incubated with 3% bovine serum albumin in PBS pH 7.4 with affinity purified rabbit anti-UPIII (P3 fraction) and AU1 (mAb) at 25°C for 1 h, rinsed three times with PBS pH7.4 at 25°C, and incubated 1 h with Alexa Fluor 594 goat anti—rabbit IgG (Invitrogen A-11037) and Alexa Fluor 488 goat anti—mouse IgG (Invitrogen A-11029). Images were collected with a Zeiss Axioskop 2 fluorescent microscopy by AxioVision 4.5 software.

### Measurement of apoptosis

Apoptosis in cultured cells was assayed using the Annexin-V-FLUOS kit (Roche Diagnostics) as previously described [30]. Annexin-positive cells were quantified by examining independent fields in 3 separate wells of a 12-well plate. For each field, brightfield and fluorescent images were captured. Total cells (brightfield) and apoptotic cells (fluorescent) were quantified manually in each image. Caspase 3/7 activity in urothelial cell cultures was determined using the Apo-One Homogeneous Caspase 3/7 Assay (Promega). Briefly, PD07siLuciferase and PD07siUPIII cultures were grown in 15 cm plates and treated with 15 ml of media containing either NU14 or NU14-1 for 3 hours (MOI 500). Dishes were washed, harvested by scraping, and resuspended in 500 µl of the manufacturer's recommended hypotonic lysis buffer. Induction of caspase 3/7 activity in each homogenate was normalized both to the levels of untreated control samples of each cell line and to total protein concentration as determined by BCA assay (Pierce Chemical). Apoptosis in bladder sections and urothelial biomemetics was detected using the TUNEL reaction (Roche) as previously described [30].

### Statistical analyses

Data were analyzed using Prism version 4.0 (GraphPad) and presented as mean±SEM. The statistical significance of differences between groups was calculated using Student's two-tailed *t* test or Mann-Whitney test for two groups or one-way ANOVA with Dunnett's post-test comparison. P<0.05 was considered significant.

## Supporting Information

Figure S1 Detecting and blocking of rabbit antisera with a monovalent Fab fragment of rhodamine-conjugated donkey anti-rabbit IgG. (A) Surface-expressed uroplakins were reacted with monospecific rabbit antisera against UPIa, followed by Alexa Fluor 488-conjugated donkey anti-rabbit IgG (a–c), or with a monovalent Fab fragment of rhodamine-conjugated donkey anti-rabbit IgG (d–f), or first with monovalent Fab fragments of rhodamine-conjugated donkey anti-rabbit IgG, and sequentially with Alexa Fluor 488-conjugated donkey anti-rabbit IgG (g–i). (B) 10 ng of biotinylated FimH/C complex was detected using HRP-labeled streptavidin. FimH (31 k-Dd) and FimC (28 k-Da) were both biotinylated.
(5.09 MB TIF)

Figure S2 UPEC bacterial strains can induce phosphorylation of UPIIIa in a FimH-dependent manner. PD07i cultures were treated for 30 minutes with NU14 or NU14-1 at an MOI of 500 or treated with PBS. Cells were washed in cold PBS followed by lysis in modified RIPA buffer. UPIIIa was immunoprecipitated and equal amounts of protein were separated using SDS-PAGE followed by immunoblotting using an anti-phosphothreonine antibody. UPIIIa from NU14-treated cells was observed to be phosphorylated to a higher degree (Lane-2) than NU14-1 (Lane-3) or saline-treated cells (Lane 1).
(0.61 MB TIF)

Figure S3 Affinity purification of UPIIIa-specific antibodies. Purified bovine AUM protein (bAUM) was separated by SDS PAGE and probed with Anti-AUM serum (A). Immunoblotting with anti-AUM serum recognized a family of bands including UPIII (migrating at approximately 45 kD) and UPIb. (B) Following stripping of the blot, AUM proteins were probed with affinity-purified, UPIII-specific antibodies (P3 fraction). P3 antibodies recognized only the family of bands corresponding to UPIIIa in various states of glycosylation.
(0.65 MB TIF)

Figure S4 UPIIIa variants are expressed on the cell surface. COS7 cells were co-infected with recombinant adenoviruses encoding UPIb and a variant of UPIb, followed by imaging by immunofluorescence. (A–D) COS7 cells expressing the UPIIIa variant T_244_A and stained with affinity-purified UPIII antibody fraction P3 (A, red channel), UPIIIa monoclonal antibody AU1 (B, green channel), DAPI (C, blue channel), or all channels combined (D). (E) COS7 cells expressing the UPIIIa variant S_282_A and stained with affinity-purified UPIIIa antibody fraction P3 (red channel), UPIIIa monoclonal antibody AU1 (green channel), and DAPI (blue). (F) COS7 cells expressing the UPIIIa variant T_244_E and stained with affinity-purified UPIIIa antibody fraction P3 (red channel), UPIIIa monoclonal antibody AU1 (green channel), and DAPI (blue). (G) COS7 cells expressing the UPIIIa variant S_282_E and stained with affinity-purified UPIIIa antibody fraction P3 (red channel), UPIIIa monoclonal antibody AU1 (green channel), and DAPI (blue). (H) COS7 cells expressing the UPIIIa variant Y_266_F and stained with affinity-purified UPIIIa antibody fraction P3 (red channel), UPIIIa monoclonal antibody AU1 (green channel), and DAPI (blue). Arrows indicate margins of cells where surface UPIIIa expression is evident.
(3.66 MB TIF)

Figure S5 UPIIIa does not mediate inflammatory responses. PD07siTNFR2 or PD07siUPIII cultures were stimulated with 10 ng/ml IL-1β or infected with NU14 (MOI 500) for 4 hours at 37°C. CXCL-8 was quantified in culture supernatants by ELISA (Pharmingen). No significant differences in CXCL-8 secretion were observed.
(0.10 MB TIF)

Figure S6 CK2 mRNA expression is reduced using RNA interference. PD07i cultures were transfected with CK2 siRNA or a non-specific control siRNA. Total RNA was purified and primers for human CK2 (forward 5′-agg cag gaa gaa agg aag gaa-3′, reverse 5′-aga cac act tcc aca aga gcc act-3′) were used to quantify CK2 mRNA expression by real-time PCR. Results were expressed as relative fold change where Ct values were normalized first to the ribosomal subunit L19 mRNA and an untreated control. Analyses were performed using the ΔΔCt method.
(0.07 MB TIF)
