# Supplementary figures and images for: Bacteria-Induced Uroplakin Signaling Mediates Bladder Response to Infection
Source: PLoS Pathog. 2009 May 1;5(5):e1000415. doi: 10.1371/journal.ppat.1000415 (PMC2669708; doi:10.1371/journal.ppat.1000415)

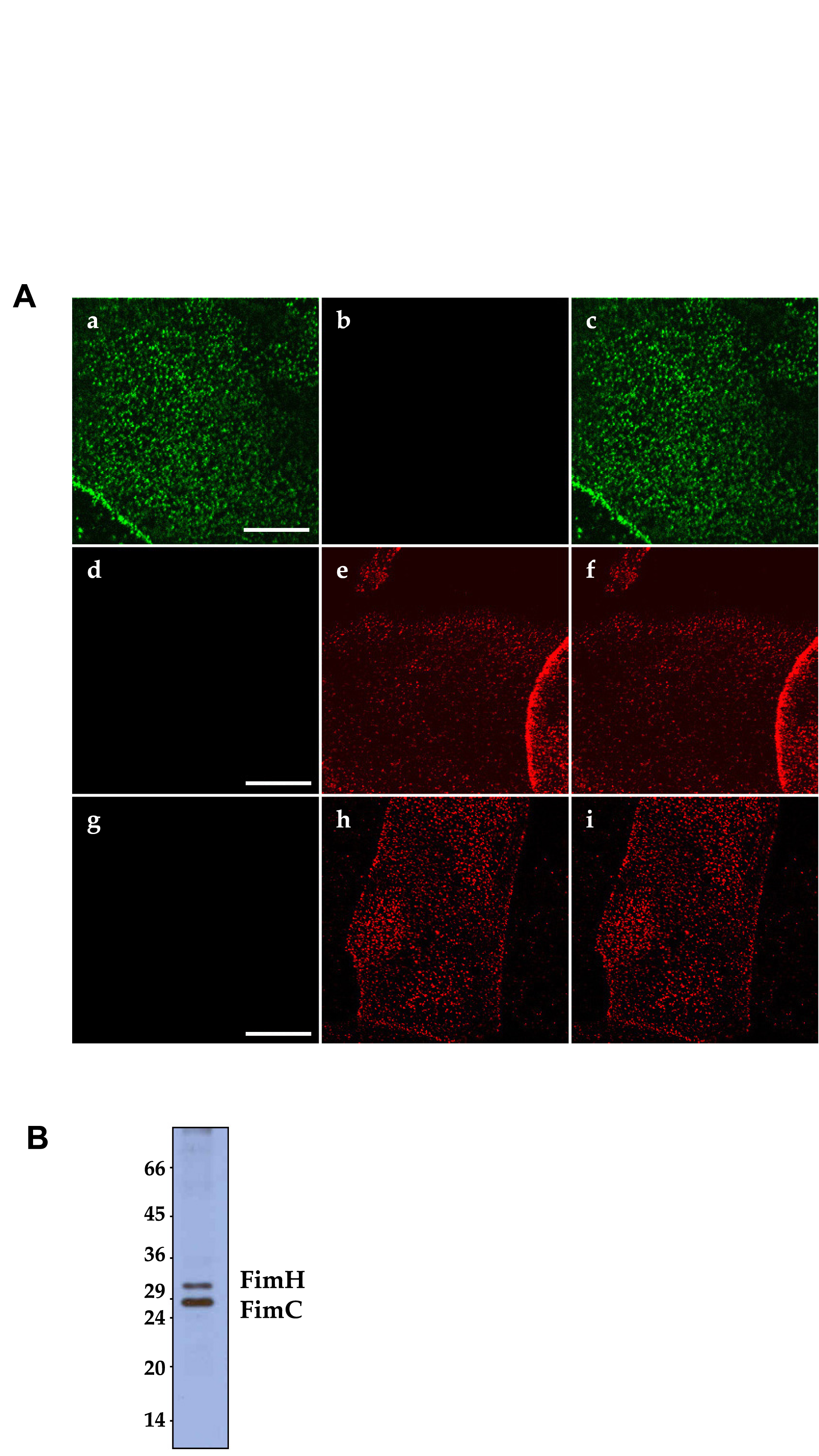

Supplement: Figure S1 — Detecting and blocking of rabbit antisera with a monovalent Fab fragment of rhodamine-conjugated donkey anti-rabbit IgG. (A) Surface-expressed uroplakins were reacted with monospecific rabbit antisera against UPIa, followed by Alexa Fluor 488-conjugated donkey anti-rabbit IgG (a–c), or with a monovalent Fab fragment of rhodamine-conjugated donkey anti-rabbit IgG (d–f), or first with monovalent Fab fragments of rhodamine-conjugated donkey anti-rabbit IgG, and sequentially with Alexa Fluor 488-conjugated donkey anti-rabbit IgG (g–i). (B) 10 ng of biotinylated FimH/C complex was detected using HRP-labeled streptavidin. FimH (31 k-Dd) and FimC (28 k-Da) were both biotinylated. (5.09 MB TIF) [file ppat.1000415.s001.tif]

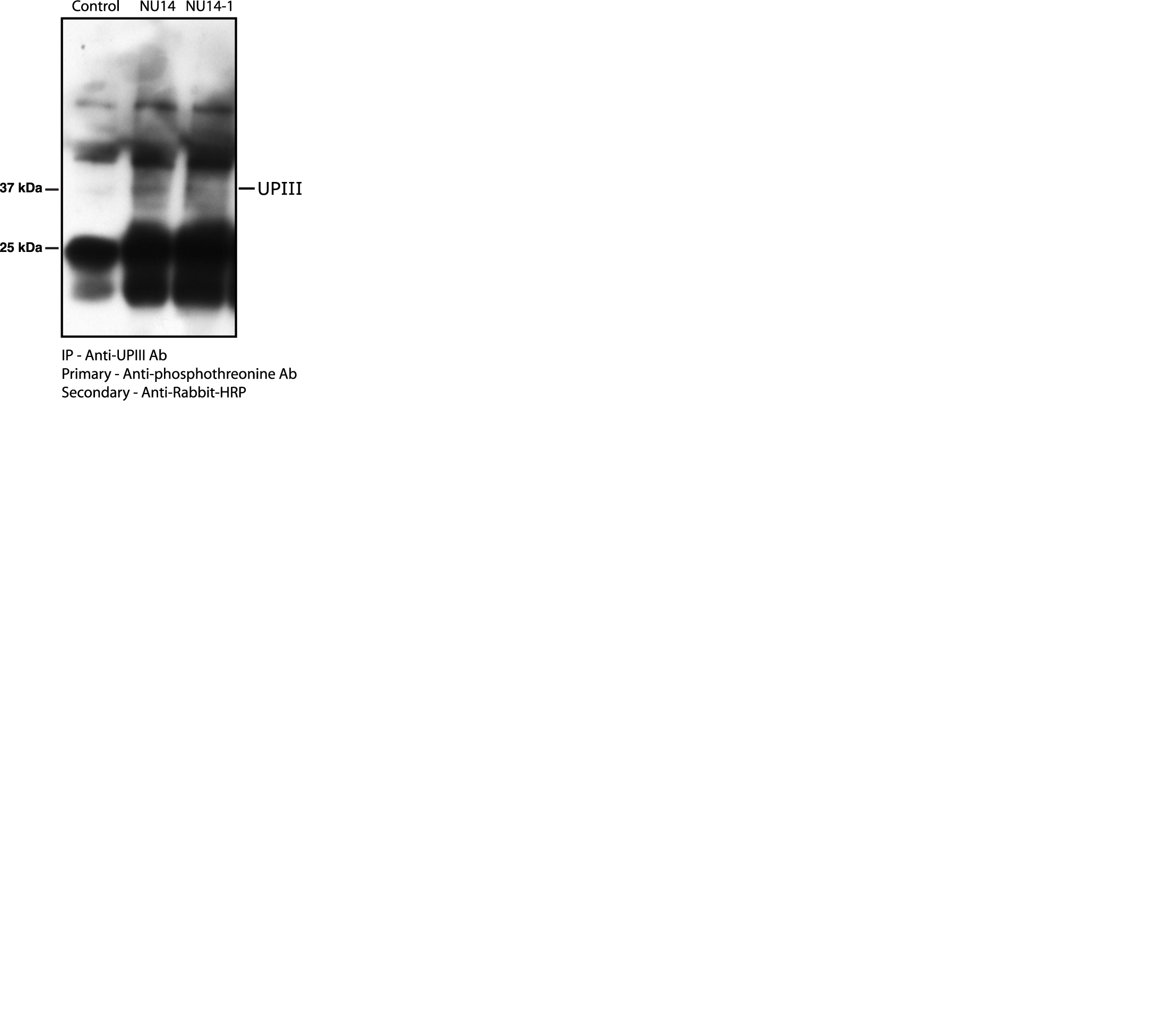

Supplement: Figure S2 — UPEC bacterial strains can induce phosphorylation of UPIIIa in a FimH-dependent manner. PD07i cultures were treated for 30 minutes with NU14 or NU14-1 at an MOI of 500 or treated with PBS. Cells were washed in cold PBS followed by lysis in modified RIPA buffer. UPIIIa was immunoprecipitated and equal amounts of protein were separated using SDS-PAGE followed by immunoblotting using an anti-phosphothreonine antibody. UPIIIa from NU14-treated cells was observed to be phosphorylated to a higher degree (Lane-2) than NU14-1 (Lane-3) or saline-treated cells (Lane 1). (0.61 MB TIF) [file ppat.1000415.s002.tif]

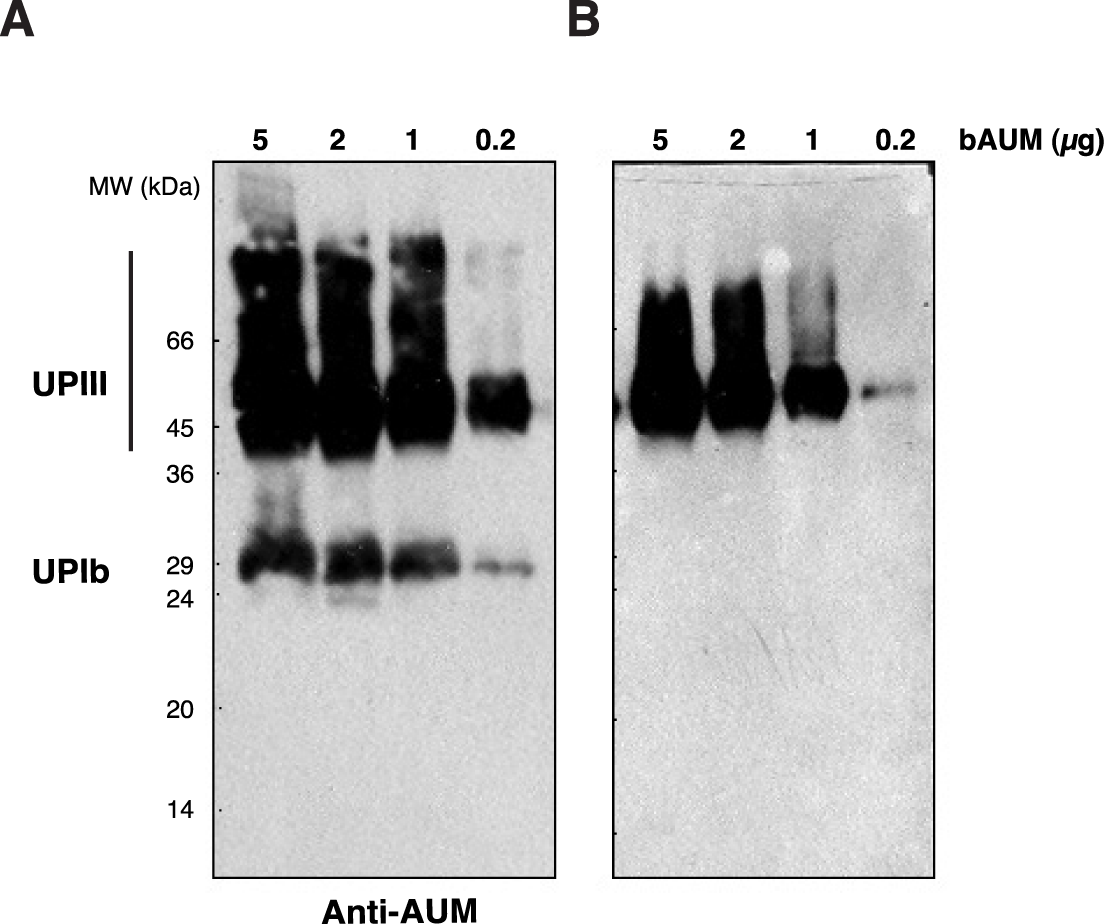

Supplement: Figure S3 — Affinity purification of UPIIIa-specific antibodies. Purified bovine AUM protein (bAUM) was separated by SDS PAGE and probed with Anti-AUM serum (A). Immunoblotting with anti-AUM serum recognized a family of bands including UPIII (migrating at approximately 45 kD) and UPIb. (B) Following stripping of the blot, AUM proteins were probed with affinity-purified, UPIII-specific antibodies (P3 fraction). P3 antibodies recognized only the family of bands corresponding to UPIIIa in various states of glycosylation. (0.65 MB TIF) [file ppat.1000415.s003.tif]

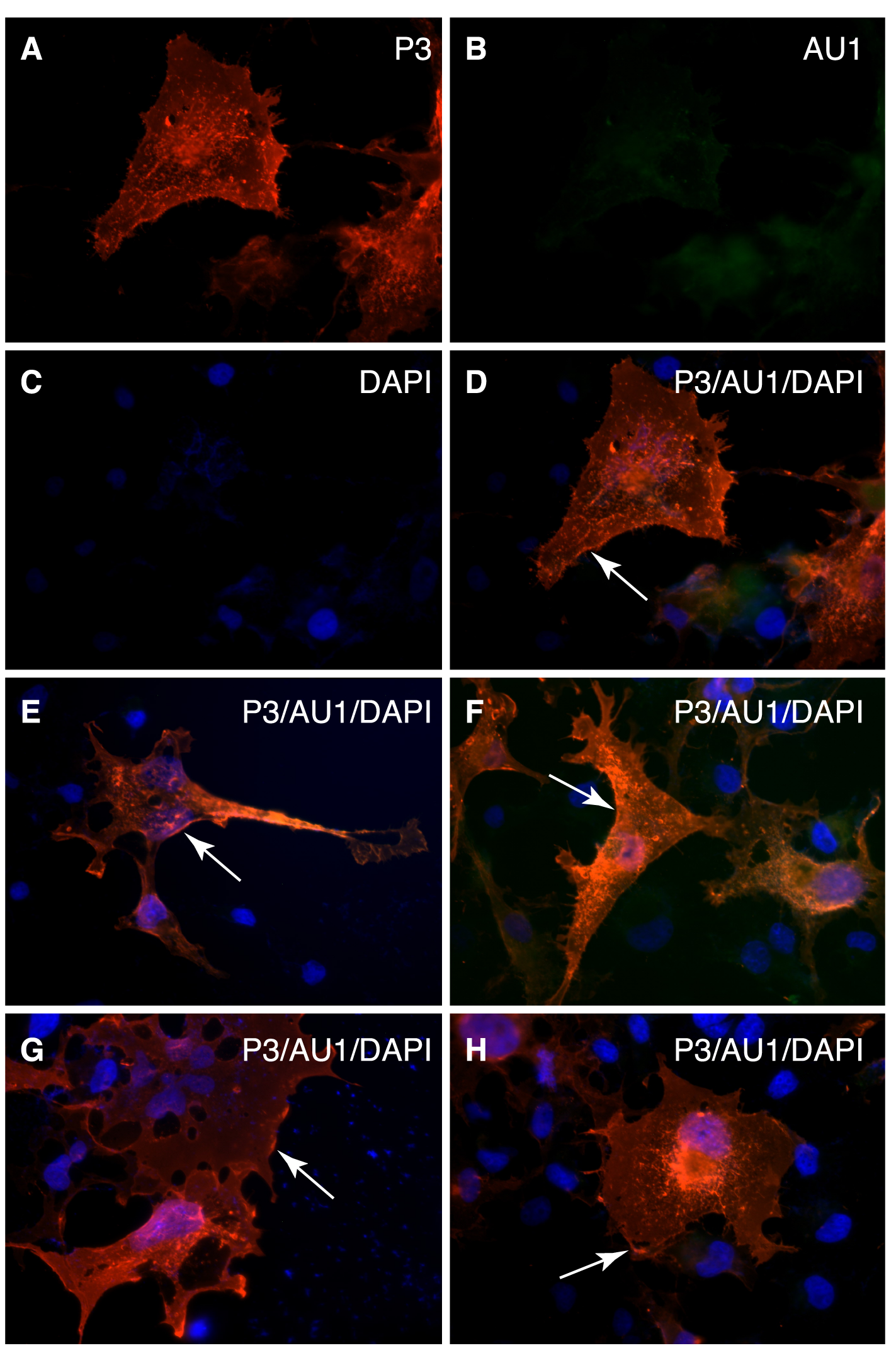

Supplement: Figure S4 — UPIIIa variants are expressed on the cell surface. COS7 cells were co-infected with recombinant adenoviruses encoding UPIb and a variant of UPIb, followed by imaging by immunofluorescence. (A–D) COS7 cells expressing the UPIIIa variant T244A and stained with affinity-purified UPIII antibody fraction P3 (A, red channel), UPIIIa monoclonal antibody AU1 (B, green channel), DAPI (C, blue channel), or all channels combined (D). (E) COS7 cells expressing the UPIIIa variant S282A and stained with affinity-purified UPIIIa antibody fraction P3 (red channel), UPIIIa monoclonal antibody AU1 (green channel), and DAPI (blue). (F) COS7 cells expressing the UPIIIa variant T244E and stained with affinity-purified UPIIIa antibody fraction P3 (red channel), UPIIIa monoclonal antibody AU1 (green channel), and DAPI (blue). (G) COS7 cells expressing the UPIIIa variant S282E and stained with affinity-purified UPIIIa antibody fraction P3 (red channel), UPIIIa monoclonal antibody AU1 (green channel), and DAPI (blue). (H) COS7 cells expressing the UPIIIa variant Y266F and stained with affinity-purified UPIIIa antibody fraction P3 (red channel), UPIIIa monoclonal antibody AU1 (green channel), and DAPI (blue). Arrows indicate margins of cells where surface UPIIIa expression is evident. (3.66 MB TIF) [file ppat.1000415.s004.tif]

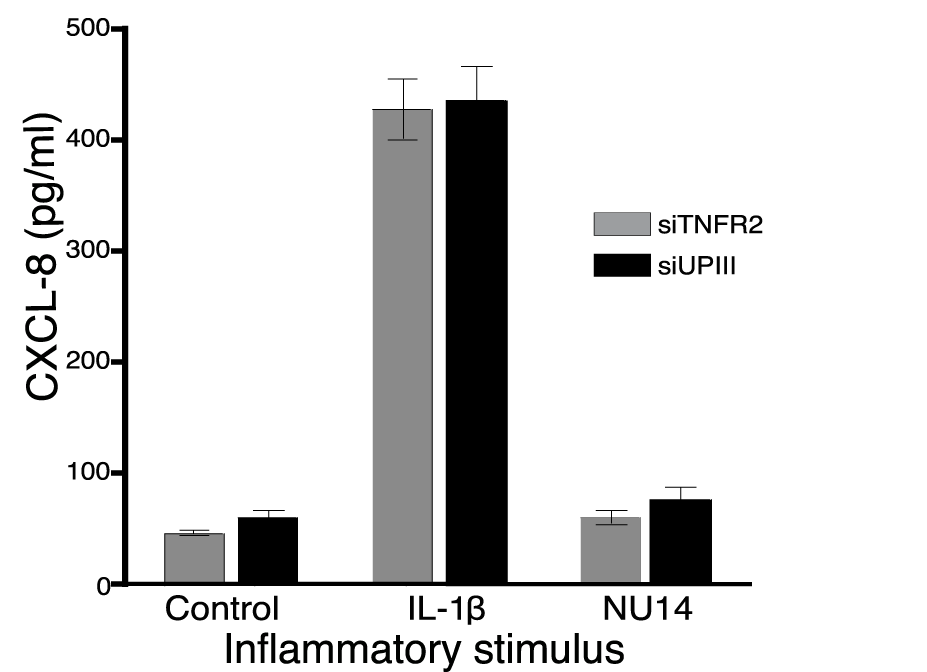

Supplement: Figure S5 — UPIIIa does not mediate inflammatory responses. PD07siTNFR2 or PD07siUPIII cultures were stimulated with 10 ng/ml IL-1β or infected with NU14 (MOI 500) for 4 hours at 37°C. CXCL-8 was quantified in culture supernatants by ELISA (Pharmingen). No significant differences in CXCL-8 secretion were observed. (0.10 MB TIF) [file ppat.1000415.s005.tif]

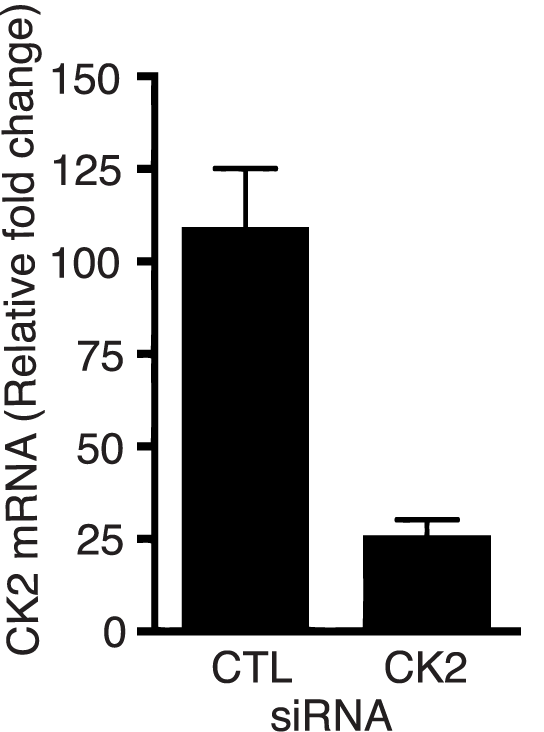

Supplement: Figure S6 — CK2 mRNA expression is reduced using RNA interference. PD07i cultures were transfected with CK2 siRNA or a non-specific control siRNA. Total RNA was purified and primers for human CK2 (forward 5′-agg cag gaa gaa agg aag gaa-3′, reverse 5′-aga cac act tcc aca aga gcc act-3′) were used to quantify CK2 mRNA expression by real-time PCR. Results were expressed as relative fold change where Ct values were normalized first to the ribosomal subunit L19 mRNA and an untreated control. Analyses were performed using the ΔΔCt method. (0.07 MB TIF) [file ppat.1000415.s006.tif]
